# Supplementary figures and images for: A Tad-like apparatus is required for contact-dependent prey killing in predatory social bacteria
Source: eLife. 2021 Sep 10;10:e72409. doi: 10.7554/eLife.72409 (PMC8460266; doi:10.7554/eLife.72409)

DZ2

NG GltJ  
(control)

NG 3108

$\Delta$ 4655  
NG 3108

$\Delta$ 4650  
NG 3108

$\Delta$ 3106  
NG 3108

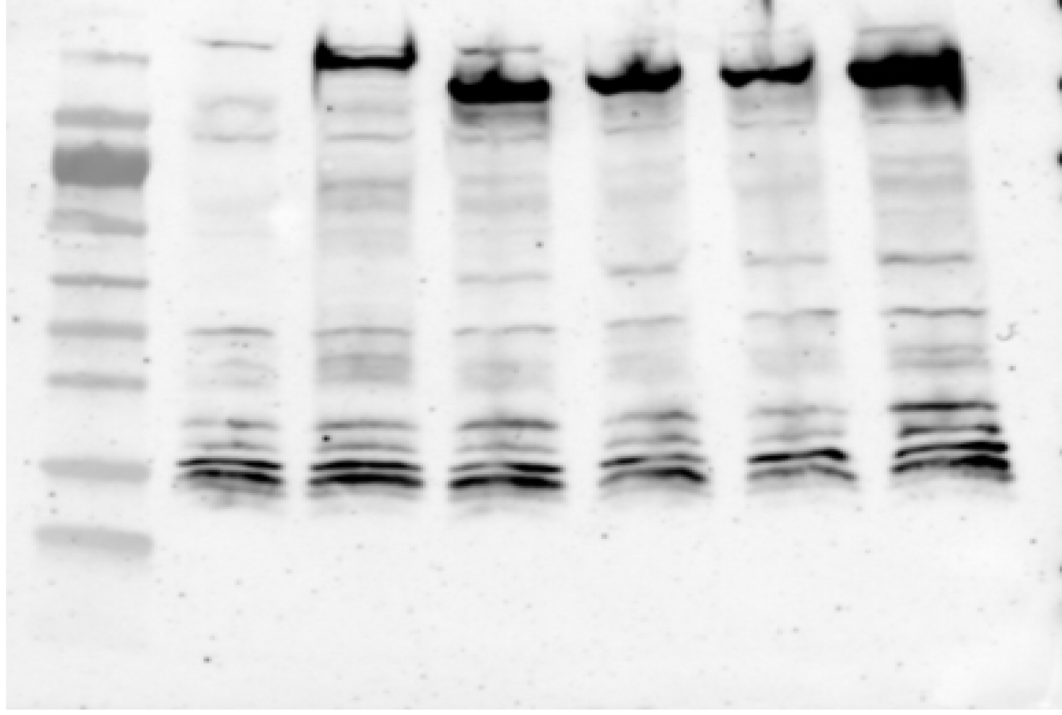

Supplement: Figure 4—figure supplement 4—source data 1. [file elife-72409-fig4-figsupp4-data1.zip › Figure 4-figure supplement 4-source data .pdf]

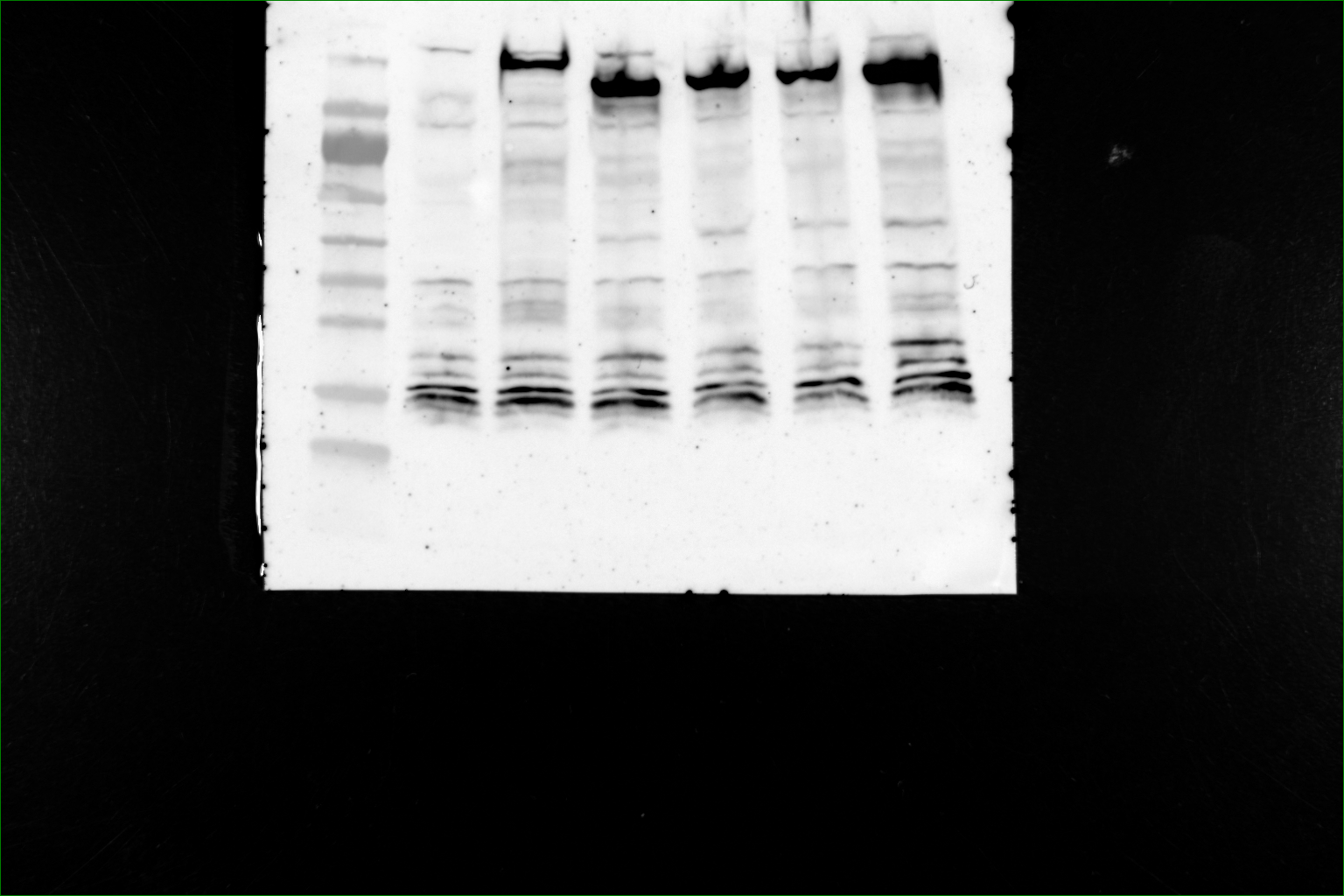

Supplement: Figure 4—figure supplement 4—source data 1. [file elife-72409-fig4-figsupp4-data1.zip › Figure 4-figure supplement 4-source data.bmp]
