# Supplementary material for: A Tad-like apparatus is required for contact-dependent prey killing in predatory social bacteria
Source: eLife. 2021 Sep 10;10:e72409. doi: 10.7554/eLife.72409 (PMC8460266; doi:10.7554/eLife.72409)
Supplement: Figure 7—source data 1. [file elife-72409-fig7-data1.docx]

Supplementary data: supermatrix alignment (used for creating the phylogenetic tree in Figure 7

Mxanthus LIEYLDLRRM----DMDRLGDDELWRRTEKAIRDIIDQMEADGELPEDV-DREELLTDVINEALGLGPLEAFLASDDISEIMVNHANQIYIERKGKLTLSEKTFSSNQAVLGVIERIVAP

Bbacteriovorus LIRTVDLKKLLVDGKQDENKEKEVREKTKREITLIV-DREAPD-VARE--ERSKLIKEVLEEALGLGPLEDLLADPDVTEIMVNGNKRVFVEKSGKVQLSPVTFTSNDHLRRIIERIVTP

Mmacrosporus LIEYLDLRRM----DMDRLGDDELWRRTEKAIRDIIDQMDADGELPEDV-DREELLTDVINEALGLGPLEAFLASDDISEIMVNHANQIYIERKGKLTLSEKTFSSNQAVLGVIERIVAP

Mstipitatus LIEYLDLRRM----DMDRLGDDELWRRTEKAIRDIIDQMEADGELPEDV-DREELLTDVINEALGLGPLEAFLASDEISEIMVNHANQIYIERKGKLTLSEKTFSSNQAVLGVIERIVAP

Saurantiaca LIEYLDLRRM----DMDRLGDEELWRRTEKAIRDIIDQMDADQELPEDV-DREELLTDVINEALGLGPLEAFLASDEISEIMVNHANQIYIERKGKLIMSEKTFSSNQAVLGVIERIVAP

Cfuscus LIEYLDLRRM----DMDRLGDDELWRRTEKAISDIIDQMEADGELPPEV-DREELLTDVINEALGLGPLEAFLASDEISEIMVNHANQIYIERKGKLVLSEKTFSSNQAVLGVIERIVAP

Dretbaense LLDMVDLSM------IDSLEPEVLKTQIRSLVTKIL-DTEERN-APLNMSERERLFSDIEDEVMGLGPLEPFLKDDTVADILVNTHNQIYVERFGKLELSESTFKDDAHLMRIIDKIVSS

Paespoeensis LIDMIDLSL------LDALGEADMRAEVARVAEGLL-WEEFQN-APLNLAERKRMLSEIQDEVIGLGPLEPYIKDPTVNDILVNGYRQVYVERAGKLELTPARFKDDNHLRRIIDRIVSL

Ppiezophilus LIDIIDLSL------LDSLSEAEMRGEIAKVTEGLL-WEEFQS-APLNLAERKRMLSEIQDEVIGLGPLEPYIKDPTVNDILVNGYKQIYVERAGKLEPTPARFKDDDHLRKIIDRIVSQ

Dtoluolica LLDIIDLSI------IDTMDKETLVKQIKHVTEKIL-REESDE-MPLNFSEREKILTELIDEVLGLGPLEPFLKDPTISDIMVNSYKKIYVERFGKIESTNARFRNDEHLMKIIDKIVSS

OsymbiontRs1 VLRMMDLKRT----DVGSMDELELKQKVEGMIDDAL-EGMAKE-IPDNI-DIADLKTSVLNEAVGLGPLEALLEDDEVTEIMVNAFDDIYVEKKGRLIRSDIAFSSDSAVMSAIERIVSP

Dsulfexigens LMNAMDLRRT----DVQAMNDDELSETVRSLVNDII-GKMARS-LPPEV-DRAKLAKEVIDEAIGLGPLEDFLAEDSITEIMVNSYDSIYYETHGKLHKSNITFSSDNAVLAVIERIVSP

Samazonensis LLKQFDLRRV----NVNEMSDIELREQSEALILHIV-GQM--A-LPETI-AATDLIREVLDETIGLGPLEALIADADVTEIMVNSHDQIFFEKSGQLYLSDISFSDDQAVLGAIERIVAP

Swoodyi LLKLMDLRRV----NVNEMSDVELRAQSESLINQII-TSI--N-LPKDI-EVESLTKEVLDETIGLGPLEGLIDDPDVTEIMVNSHDQIFYEKAGNLYLSDIAFSDDQAVLGAIERIVTP

Bgladioli LIAELDLRRL----NVARMDDDELRRTVGGALDEIL-DNDAAF-RSADV-SLEALRKSVFDEVIGLGPLEELIADPEVSEIMVNCHDEIFVERRGKLTRSRVIFTDDRAVLGAIERIVAP

Bsp.CCGE1001 VIAALDLRRL----NVARMEDDELRKTVGAALDDIL-NHDPMF-RTPDI-PLDVLKKSVFDEIIGLGPLEELIADPSVSEIMVNCHNEIFVEQDGQLTRSPVIFTDDRAVLGAIERIVAP

Sloihica LLKQMDLRRV----NVNEMSDTELRAQSEVLLKQII-DNF--K-LPDDI-EISALTKEVLDETIGLGPLEGLIADPDVTEIMVNSHDQIFYEKAGNLYLSDIAFSDDQAVLGAIERIVTP

Aferrivorans VRSQMDLRRV----DLGALSLEELRRMVGELVREVV-WRMN---PPLEI-DRQRLVKEVLDEAVGLGPLEDFLTDATITEIMVNRFDEVFIERQGKLLAAPALFSSEMAVRHIIDRIVAP

Pxenovorans VIAALDLRRL----NVARMEEDELRKTVSAALDDIL-NHDASF-HTADI-PLSALKKSVFDEIIGLGPLEELIADPTVSEIMVNCHNEIFIEQNGQLTRSPVIFTDDRAVLGAIERIVAP

Csp.GA3-3 LIAALDLRRV----NVARMAEDELCELVGSLLDDIL--KESVF-RSQEI-PLDALKQSVFDEVIGLGPLEELLDDDAVSEIMVNCHDEIFVERAGRLTRSPVIFTDDRAVLGAIERIVAP

Tsp.28 LLDAIDLRRR----DLVRMSDDELRAEAESLLVELI-D-KETA-LPDGI-DRDSLRRDVLNEAVGLGPLDELLADEAISEIMVNRHDEIFLERHGRLERHPGSFTSERAVMGIIERIVTP

Cnecator LIAALDLRRV----NVARMAEDELCELVGSLLDDIL--KESVF-RSQEI-PLDALKQSVFDEVIGPGPLEEPLDDDAVSEIMVNCHDEIFVERAGRLTRSPVIFTDDRAVLGAIERIVAP

Tterpenica LLDAIDLRRR----DLVRMSDGELRAETEALLIELI-D-QEAA-LPEAI-DRDRLRRDVLNEAVGLGPLEALLEDDSITEIMVNRHDEIYIERRGRLERHPASFTSERAVLGVIERIVTP

Acaldus VRAQMDLRRM----DLSSMSREDLQRFVSNLAAEVL-QGLR---LPPEL-DRERLIKEVVDEAVGLGPLEDLLADETVTEIMVNRFDEVFIERRGRLEPAPTVFSSEQAVRNIIDRIVAP

Veiseniae LIEAMDLRRH----DVSRMSDEHLRAEADRLIVKIM-QDMAAD-IPAAL-EHGALRKQILDEAVGLGPLEELLADDSVTEVMVNRFDEIYIERAGRLQKHALSFTSDQAVMGVIERIVAP

Hneapolitanus LVKQMDLRRV----DVHGMSDDELRAKTGALIDEII-QREFSD-LPASI-DRSRLAKEVLDEAIGLGPLEDLISDDSVTEIMVNRADEIFVERAGRIERSPVIFTSDKAVLSVIERIVAP

Phospita VIAALDLRRL----NVSRMADDELRTTVSDALDDIL-ARDASF-RRPGI-PLPALKKSVFDEIIGLGPLEELIADPTISEIMVNRHDEIFVEQQGRLVRSPVIFTDERAVLGAIERIVAP

Dacidovorans LIDTMDLRRH----DVSSMSDEHLRAEADRLIARTL-QDMDDE-IPAQL-DRNALRRQVLDEAVGLGPLEELLADETVSEIMVNRFDEIYVERAGRLQKHALSFTSDRAVMGVIERIVAP

Taminoaromatica LLDTIDLRRR----DLNRMSDDELRAETTALVREII-A-AEAA-LPGGL-DREQLCSEVLDEAIGLGPLETLLTDESVSEIMVNRFDQIFVERGGRIAPHPTTFTSDRAVLGVIERIVAP

Smaltophilia LVKQMDLRRL----DVRSMDDESLRNATIKLIDEVM-QREFSE-LPKTI-NPRRLAKEVLDEAIGLGPLEDLIDDATVTEIMVNAHDQIFIERAGRIERSPIVFTSDRAVLSAIERIVTP

Pphymatum VIDALDLRRL----NVSRMADDELRATVGDALDDIL-ARDASF-RRSDI-PLPTLKRSVFDEIIGLGPLEELIADPTISEIMVNRHDEIFVEQQGRLRRSPVIFTDERAVLGAIERIVAP

Ahongdengensis LVDYMDLRRV----NVADMSDQELRNFTAGAIDKIIRDQN--D-IDAEI-DQEELARQVLAEAVGLGPLEPLLADESISEIMVNARDEIYYERNGRLNQSRVTFTDDTSVISAIERIVSP

Rspathiphylli LVRQMDLRRV----DVRGMTDDDLRQKTSSLIEEII-EREFADELPRTI-NRRRLAKQVLDEAIGLGPLEDLLADETVTEVMVNCSDDIYIERAGRIEKSEVIFTSDKAVLAAIERIVAP

Psp.T7-7 LLEALDLRRR----DIASMSDEALRSEASAALSDII-S-TDTE-LPAYI-DKPALLIEVVNEAVGLGPLEPLLADQAVTEIMVNRHDEIFVEINGQLKRHTAAFSSEQAVLGVIDRIVSP

Vparadoxus LLEQLDNRRI----DTAQMKDEELRSNVRALISEII-R-DSRE-LPADI-DRTLLSQQLLDEAIGLGPLEVLLKDESVSEVMVNRFDEIWVERSGRLERTNVAFTSNIAVLGAIERIVTP

Amarplatensis LLEGLDLRRR----DVAGMSDGTLRAEAERLLTQIV-A-KDAD-LAGDV-DKAALCREVLDEAVGLGPLEPLLAAPDITEIMVNRYDEIYVERAGRLWRHPAAFTSEQSVRWVIERIVTP

Aarsenitoxydans LLSALDLRRR----DVAGMSDGTLRAEAERLLTHIV-A-ADSE-LPADA-DRVSLCRDVLDEAVGLGPLEPLLAAADVTEIMVNRYDEIYVERGGRLWRQQAAFTSEQSVRWVIERIVTP

Axylosoxidans LLGALDLRRR----DVSGMSDATLRAEAARLLAQIV-A-DEDV-SAPGV-DRAALCREVLDEAVGLGPLEPLLAAPDITEIMVNRHDEIYVEREGRLWRHAAAFTGEQSVRRVIERIVTP

Dvulgaris LLEMMDLAA------AESLPPDRLAAEISRLVEKLL-REEFRQ-APLNAQEQRQLVEDIRDEVMGLGPLEPLLRDPTVNDILVNNYRMVYVERRGKLIRVNTRFLDDDHLRKIIDRIVAR

Bexovorus LIRTVDLKKILAEVGDNESKEKELRAKTQRDIGLIV-DREAPD-LPRD--ERQRLVKDVLEEALGLGPLEDMLADPSISEIMVNGANRIFIEKGGKVQLSGIKFTSNDHLRRIIERIVTP

Delongatus LVEELDIAS------ITELPREALTASIRNHLEQI---TRARN-LPLNRQERLDLVADLMDEILGLGPIEPLIQDETVQDILVNGFNQVFVERQGMLEHTDIQFRDNDHLMQIVDRIVAG

Hmarinus LVDEMDLKNS---DDNDPKAAIIMREQTKKMVVELLGKEDTKS-VFQTREDMNRIVKEILDEALGLGPLEDLLADKECSEIMVVGPDKIFYEKSGKVKKSDITFTNDRQVLNVIERIVAP

Bstolpii LVEEMDLKKA---DDNDPKAQIILKEQTKKVVVELLGKEDTKG-ILNTREDMNQIVKEILDEALGLGPLEDLLRDKSISEVMVVGPYKIYYEQGGKIKLSEITFTNDRQVLNVIERIVAP

Dphenolica LLDIIDLSI------IDTMDKETLVKQIKHVTEKIL-REESDE-MPLNFSEREKILTELIDEVLGLGPLEPFLKDPTISDIMVNSYKKIYVERFGKIESTNARFRNDEHLMKIIDKIVSS

Dcetonica LVEQLDFAS------LTELPKEALTGSIRASLEQI---TTAHH-LPLNQKERADLIADLMDEILGLGPIEGLVQDETVQDILVNGHDQVFVERRGILEKTDVQFRDDDHLMQVIDRIVSA

Dmultivorans LLDRIDLSV------IENVEKNVLKAKLRSVIEELV-REDGAG-VPLNFAERERLFQEIQDEVIGLGPLEPFLQDPTVSDILVNTYERIYVERFGKLEPSDARFKDNDHLKRIIDKIVSA

Cvibrioides LLNTIDLSQ------LAQLDQKAAAEEIRDIVAEL---VAIKN-VSMSVAEQEHLVQDIINDVLGYGPLEPLLARDDIADIMVNGAHRVFIEVAGKVQLTNVRFRDNLQLMNICQRIVSQ

Atumefaciens LIDTIDLSQ------LAKLDTESAREEIRDIVNDI---ITIKN-FAMSISEQEELLEDICNDVLGYGPLEPLLARDDIADIMVNGSGQTFIEVNGKTIESDIRFRDNAQLLSICQRIVSQ

Aexcentricus LLNTIDLSQ------LAQLDQKAAAEEIRDIVSEL---VTIKN-VSMSNAEQDALVQDIINDVLGYGPLEPLLARDDIADIMVNGAGRVFIEVSGKVQLTNVRFRDNAQLMNICQRIVSQ

Bdiminuta LMNTIDLAQ------LAQLDQKAASEEIRDIVAEL---VAIKN-VSMSVAEQEHLVQDILNDVLGYGPLEPLLARDDIADIMVNGAGRVFIEVGGKVQLTNVRFRDNGQLMNICQRIVSQ

Mmaris LIDTIDLGQ------LARLDNETAAEEIRDIVTEI---ISIKN-VAMSIAEQEQLLQDICNDVLGFGPLEPLLARDDIADIMVNGADKVFIEVNGKLEETNIRFRDNAQLMNICQRIVSQ

Plavamentivorans LIDTIDLTQ------LAQLDSESAREEIRDIVNEI---LSIKN-VVMSISEQEALLQDICNDVLGYGPLEPLLARDDIADIMVNGCERTFIEVNGKVELTNIRFRDNSQLMNICQRIVSQ

Nhamburgensis LIEAIDLAQ------LAKLDGESAREEIRDIVNEI---IAIKN-IVMSIAEQEELLDDICNDVLGYGPLEPLLSRDDIADIMVNGAGTVFIEVAGKIQRTGIRFRDNQQLLNICQRIVSQ

Amanganoxydans LIDTIDLSQ------LARLDAESAREEIRDIVNDI---VAIKS-FAMSISEQEDMLSDICNDVLGYGPLEPLLARDDIADIMVNGARQTFIEVAGKVLETNVRFRDNQQLLNICQRIVSQ

BbacteriumSG-6C LIEAIDLAQ------LSKLDVESAREEIRDIVNEI---IAIKN-IVMSIAEQEELLDDICNDVLGYGPLEPLLARDDIADIMVNGAGTVFIEVGGKIQKTGIRFRDNQQLLNICQRIVSQ

Aclevelandensis LIEAIDLAQ------LSKLDVESAREEIRDIVNEI---IAIKN-IVMSIAEQEELLDDICNDVLGYGPLEPLLARDDIADIMVNGAGTVFIEVGGKIQKTGIRFRDNQQLLNICQRIVSQ

Lsediminis LKERLDLHSF----QITDYSSPRVIRQVSEKLKELI-SRRQRE-IPPPY-TPDEVFKEMMDEVCGLGPIEDLVKHKGVSEIMVVDREHIYAELNGKIVLTDRIFNDEKSMMTVIERIVIP

Llitoralis LKERLDLHSF----QITDYSSPRVVRQVSEKLKELI-ARRQRE-IPRPY-TPDQVFKEMMDEVCGLGPIEDLVKHRGVSEIMVVDREHIYAELNGKIVLTDRIFNDEKSMMTVIERIVIP

Pcaeni LKERLDLHSF----EITDYRTPRTVRQVSERLKQLL-EIHAHE-IPPPF-TRDDVFKELMDEVCGLGPIEDLIKHRKVSEIMVVDRDHIYAELDGNIVLTDRFFNDEKSMMTVIERIVIP

Bsediminis LKERLDLHSF----EITDYRTPRTVRQVSERLKQLL-EIHAHA-IPPPY-SRDDVFKELMDEVCGLGPIEDLIKHRKVTEIMVVDRDHIYAELNGDIVLTDRFFNDEKSMMTVIERIVIP

: .* : .: : * **:: : :::* : * * * .: : ::**

Mxanthus IGRRIDESSPLVDARLKDGSRVNAIIPPLALKGPCITIRKFKKDSLKIADLIKYKTVTAQMAEFLEMCVKARRNIVISGGTGSGKTTTLNIISSFIPEGERIITVEDAAELQLPQDHWVQ

Bbacteriovorus LGRQINDSTPYVDARLKDGSRVNAVIEPLAIDGPALTIRKFKKGGITAEKYIGYGSITKNMIDFLRICVENGLNVVISGGTGSGKTSLLNMLSSFIPSNERVITVEDAAELQLQQEHVVR

Mmacrosporus IGRRIDESSPLVDARLKDGSRVNAIIPPLALKGPCITIRKFKKDSLKISDLIKFKTVTAQMAEFLEMCVKARRNIVISGGTGSGKTTTLNIISSFIPEGERIVTVEDAAELQLPQDHWVQ

Mstipitatus IGRRIDESSPLVDARLKDGSRVNAIIPPLALKGPCITIRKFKKDSLKIQDLIKYKTLTAQMAEFLEMCVKARRNIVISGGTGSGKTTTLNIISAFIPDSERIVTVEDAAELQLPQDHWVQ

Saurantiaca IGRRIDESSPLVDARLKDGSRVNAIIPPLALKGPCITIRKFKKDSLKIQDLIKYKTITAQMAEFLEMCVKARKNIVISGGTGSGKTTTLNIISSFIPDDERIVTVEDAAELQLPQDHWVQ

Cfuscus IGRRIDESSPLVDARLKDGSRVNAIIPPLALKGPCITIRKFKKDSLKIQDLIKFKTVTAQMAEFLEMCVKARKNIVISGGTGSGKTTTLNIISSFIPEDERIVTVEDAAELQLPQDHWVQ

Dretbaense VGRRIDESSPMVDARLADGSRVNVIIPPLALDGPVMSIRRFGKDPLKMDDLIMLRAFTQGIGEIMKGIVRSELNVVISGGTGSGKTTLLNCLSQFIPATDRIITIEDAAELQLKQEHVVR

Paespoeensis VGRRIDESQPLVDARLLDGSRVNAIIPPLAIDGPSLSIRKFSKDPLETSDLIAFNSLTQPMAELMDGIVKARLNVVISGGTGSGKTTLLNCLSRSIPEDERIVTIEDAAELQLKQDHVVR

Ppiezophilus VGRRIDESQPLCDARLMDGSRVNAVIPPLAIDGPSLSIRKFSKDPLEISHLISFNSLTEPMARLMDGIVKARLNVLISGGTGSGKTTLLNCLSRNIPEDERIVTIEDAAELQLKQDHVVR

Dtoluolica IGRRIDESNPMVDARLADGSRVNVIIPPLALDGPMVSIRRFSVVPLELDDLIKNQTLVPEFRSILEGLVQSRLNILISGGTGSGKTTLLNVLSRFIPETERIVTIEDAAELQLKQEHLVR

OsymbiontRs1 LGRRIDESSPMVDARLPDGSRVNAIIPPLALRGPTLTIRKFSKKKLNVDDLIGFGTINQAMAHFLQMAVEQHCNIVISGGTGSGKTTLLNIMSNFIPVTERIITIEDAAELKLAQPHVVS

Dsulfexigens LGRRIDESSPMVDGRLKDGSRINAIIPPLAIKGPCLTIRKFSRKKLSIDDFVQFGSISNKMVDFLEKAVHYRKNIIISGGTGSGKTTFLNVLSDFIPHDERIITVEDSAELKLGQPHVVS

Samazonensis IGRRIDEGSPMVDARLKDGSRVNAVIPPLALKGPCITIRKFMQRRLGASDLVGFGSMSQHMADFLEMAVKQKCNVVISGGTGSGKTTLLNVLSNFIPEDERIITVEDAAELRLYQPNLVS

Swoodyi IGRRIDESSPMVDARLKDGSRVNAVIPPLALKGPCITIRKFMQQRLSCDDLVKFGSMNQAMAGFLETAVNQKKNIIISGGTGSGKTTLLNVLSNFIPDNERIVTVEDAAELQLYQPNLVS

Bgladioli IGRRIDESSPMVDARLSDGSRVNAVIPPLALKGPSITIRKFSQRKLTGEDLIDFGSMSADMLAFLRTAVEQRANIIISGGTGSGKTTLLNVLSNYIPDDERIVTVEDAAELQLSQPNLVA

Bsp.CCGE1001 IGRRIDESSPMVDARLADGSRVNAVIPPLALKGPSITIRKFSRRKLVGEDLIRYGTLSPHMLEFLHTAVKQGANIIISGGTGSGKTTLLNVLSSYIPDDERIVTVEDAAELQLSQPNLVS

Sloihica IGRRIDESSPMVDARLKDGSRVNAVIPPLALKGPCITIRKFMQRRLSCNDLVAFGSMSQAMAEFLEIAVKQKRNVVISGGTGSGKTTLLNVLSNFIPDNERIVTVEDAAELQLYQPNLVS

Aferrivorans IGRRIDESSPLVDARLADGSRVNAVIPPLSLKGACITIRKFSKKRLQMEDLMAYGSIEARMAHFIQVCVQQRKNIIISGGTGSGKTTLLNVLSNYIPDHERIVTIEDAAELRLYQPNLVS

Pxenovorans IGRRIDESSPMVDARLSDGSRVNAVIPPLALKGPSITIRKFSRQKLGGEDLIRFGSMSPDMLEFLHTAVKEGANIVISGGTGSGKTTLLNVLSSYIPDDERIVTVEDAAELQLSQPNLVS

Csp.GA3-3 LGRRIDESSPMVDARLPDGSRVNAVIPPLALKGPNITIRKFSRKKLRGEDLVGFGSLSPEMLEFLRTAVERRANIVISGGTGSGKTTLLNVLSSFIPDDERIVTVEDAAELQLSQPNLVS

Tsp.28 IGRRIDEASPMVDARLKDGSRVNAIIPPLAIKGPALTIRKFGRKLFSHADLVALGAMSAQMAEFLRVCVQQRRNVLISGGTGSGKTTLLNVLSNFIPDGERIITIEDAAELRLAHANLIS

Cnecator LGRRIDESSPMVDARLPDGSRVNAVIPPLALKGPNITIRKFSRKKLRGEDLVGFGSLSPEMLEFLRTAVERRANIVISGGTGSGKTTLLNVLSSFIPDDERIVTVEDAAELQLSQPNLVS

Tterpenica IGRRIDESSPMVDARLKDGSRVNAVIPPLAIKGPALSIRKFGRKVLTDADLIAFGAMSTQMAAFMRICVEQRKNILISGGTGSGKTTLLNVLSNFIPDGERIITIEDAAELRLAHSHLIN

Acaldus IGRRIDESSPLVDARLHDGSRVNAVIPPVALKGANITIRKFSKKRLQMEDLIGFGSVEPRMAEFLRVAVEQRKNIIISGGTGSGKTTLLNVLSNFIPPNERVVTIEDAAELKLYQPNLVS

Veiseniae LGRRIDESSPMVDARLKDGSRVNAIIAPLALKGSTLTIRKFAKRKLDAGDLVQFGSLSPAMADFLRICVQARKNIIVCGGTGSGKTTLLNILSNFIPPGERVITVEDAAELRLNHEHLIS

Hneapolitanus LGRRIDESSPMVDARLLDGSRVNAVIPPVALRGPSLSIRKFSKRKMTGQDLLDFGSCNAEMLEFIEVAVREKKNIVVTGGTGSGKTTLLNILSNFIPDHERIVTIEDAAELKLTQPNLVS

Phospita IGRRIDESSPMVDARLKDGSRVNAVIPPLALKGPSMTVRKFPQHKLRGDDLQTFGSLSHAMLAFLRTAVEHRANIVISGGTGSGKTTLLNVLSDYIPDAERIVTVEDAAELQLSQPNLVA

Dacidovorans LGRRIDESSPMVDARLKDGSRVNAIIAPLALKGSTLTIRKFARRKLNAADLVQFGALSPAMADFLRICVEARKNIIVSGGTGSGKTTLLNILSNFIPSGERVITVEDAAELKLAHEHLIS

Taminoaromatica LGRRIDESSPMVDARLRDGSRVNAIIPPLALKGPTLTIRKFARRALEVADLVRMGSLSHEMAAFLRTCVEQRRNIVVSGGTGSGKTTFLNLLSNFIPDGERILTIEDAAELRLRHSHLVS

Smaltophilia LGRRIDESSPMVDARLKDGSRVNAIIPPVALRGPSISIRKFAKRKLEGKDLLTFGSLSQPMLEFLIVAVRERRNIVVTGGTGSGKTTLLNILSNFIPDTDRIVTIEDAAELKLVQPNLVA

Pphymatum IGRRIDESSPMVDARLKDGSRVNAVIPPLALKGPSMTVRKFPQQKLRGYDLLGFGSLSPAMLAFLRTAVEQRANIVISGGTGSGKTTLLNVLSDYIPDEERIVTVEDAAELQLSQPNLVA

Ahongdengensis LGRRIDESSPMVDARLKDGSRVNAVIPPLALKGPNITIRKFMKERLTAQHMRQFGSLSAEMVDFLELAVRNRKNVVISGGTGSGKTTLLNVLSNFIPDSERIITVEDAAELKLYQPNLVS

Rspathiphylli LGRRIDESSPMVDARLADGSRVNAVIPPVALRGPSLSIRKFSKRKLSGDDLLQYGSVNRQMLEFMQVAVRERRNIVVTGGTGSGKTTLLNILSNFIPDHERIVTIEDAAELRLSQPNLVA

Psp.T7-7 LGRRIDESSPMVDARLRDGSRVNAVLAPVALRGSSLTIRKFPQKRPDMNDLLRLEAFDESMQIFLAESVRSKKNIIVSGGTGSGKTTLLNVLSNCIPRNERIVTIEDAAELRLEHPHLVA

Vparadoxus LGRRIDESSPMVDARLKDGSRVNAIIPPLALRGPSVTIRKFPKNRMGHEGMIRTRSITPQMVEFMRIAVEEKLNIIVSGGTGTGKTTLLNMVSNFIPANERIVTIEDAAELSLGQPNLVS

Amarplatensis LGRRIDESSPMVDARLTDGSRVHAIIPPVAMKGASLTIRKFPQRRPKMADLIAAVSLSEGMAQFLALCVRMRKSLVVSGGTGSGKTTLLNILSNEIPDGERVVTIEDAAELRLNHNHLVA

Aarsenitoxydans LGRRIDESSPMVDARLPDGSRVHAVIPPVAMKGASLTIRKFPRQRPQMPDLIALGALSNPMAQFLALCVRMRKNLVVSGGTGAGKTTLLNILSNEIPDGERVVTIEDAAELRLNHGHLVG

Axylosoxidans LGRRIDESSPLVDARLPDGARVHAVIPPVALKGASLTIRKFPRSRPDMPALLAAGALSDAMARFLARCVRLRKNLLVSGGTGSGKTTLLNVLSNEIPEGERVVTIEDAAELRLNHAHLVA

Dvulgaris IGRRVDEASPMVDARLADGSRVNAIIPPLALDGPSLSIRRFSKDPLELEDLIRFGALTPEMGEVLRGIVKARLNIIVSGGTGSGKTTMLNCLSRFVPHDERIVTIEDAAELQLKQDHVVR

Bexovorus LGRQINNATPYVDARLKDGSRVNAVIEPLSLDGPALTIRKFKKGGISAEKYIEFGSATKNMLDFLRISVEYGYNVVISGGTGSGKTSLLNMISQFIPAHERVITVEDAAELQLMQEHVVR

Delongatus VGRRVDESSPMVDARLPDGSRVNVIIPPLALDGPVVSIRKFGRTPVSLENLLATKACTPEMDRFLRAAVRSKLNVLISGGTGAGKTTLLNILSGYIPPAERIVTIEDSAELRLNQPHVVK

Hmarinus IGRRIDEKTPYVDARLKDGSRVHAIIPPSALDGCCITIRKFPEEVVTYKDYIKWGSLTQNMADFLRIAVEGHRNIVVSGGTGSGKTTLINILGGFIPANERIITCEDSAELNFPQDHVVR

Bstolpii IGRRIDEKTPYVDARLKDGSRVHAIIPPSAIDGCSITIRKFPEKRLTYKDLVKFGSMTENMADFLRIAVEAHRNIIVSGGTGSGKTTLINVLGGFIQSNERIITCEDSAELNFPQEHIVR

Dphenolica IGRRIDESNPMVDARLADGSRVNVIIPPLALDGPMVSIRRFSVVPLELDDLIKNQTLVPEFRSILEGLVQSRLNILISGGTGSGKTTLLNVLSRFIPETERIVTIEDAAELQLKQEHLVR

Dcetonica VGRRVDESSPMVDARLPDGSRVNVIIPPLALVGPVVSIRKFGRHPVTIDQLLDSLALTDEMNAFLKAAVRTKLNILISGGTGAGKTTLLNVLSGYIPLAERIVTIEDSAELRLNQPHVVG

Dmultivorans VGRRIDESSPMVDARLPDGSRVNAIIPPLAIDGPILSIRRFSVDPLEMNDLLSYKTLTPQIAEILQACVRAKLNILISGGTGSGKTTTLNVLSRYIPKNERIVTIEDSAELQLKQDHVVR

Cvibrioides VGRRVDESSPICDARLPDGSRVNVIAPPLALDGPTLTIRKFKKDKLTMKNLVEYASISPEGARVLGVIGACRCNVIISGGTGSGKTTLLNTMTAFIDPTERVVTCEDAAELQLQQPHVVR

Atumefaciens VGRRVDESSPICDARLPDGSRVNVIAPPLAIDGPALTIRKFKKDKLTLDQLVRFGAITPEGAVLLQIIGRVRCNVVISGGTGSGKTTLLNCLTSYIDKTERVITCEDTAELQLQQPHVVR

Aexcentricus VGRRVDESSPICDARLPDGSRVNVIAPPLALDGPTLTIRKFKKDKLTMRNLVEFGSISPEGARVLGVIGASRCNVLISGGTGSGKTTLLNTMTAFIDPTERVITCEDAAELQLQQPHVVR

Bdiminuta VGRRVDEASPICDARLPDGSRVNVIAPPLAIDGPTLTIRKFKKDKLTMRNLVEYASISPEGARVLGVIGASRCNLVISGGTGSGKTTLLNTLTAFIDPTERVITCEDAAELQLQQPHVVR

Mmaris VGRRVDESSPICDARLMDGSRVNVIAPPLALDGPTLTIRKFKKDKLQMQNLVEFGSITPEGAKVLSIIGASRCNVLISGGTGSGKTTLLNCMTGFIEEDERVVTCEDAAELQLQQPHVVR

Plavamentivorans VGRRVDESSPICDARLPDGSRVNVIVPPLAIDGPALTIRKFRRDKLMMNDLVQYNSISQEGAEVLGIIGKVRCNVLISGGTGSGKTTLLNCLTGFIDTDERVITCEDAAELQLQQPHVVR

Nhamburgensis VGRRVDESSPICDARLADGSRVNAIVPPLAIDGPALTIRKFRKDKLTLDQLVKFGAITPEGGTILQIIGRCRANVLISGGTGSGKTTLLNCLTNYIDHDERIITCEDAAELQLQQPHVVR

Amanganoxydans VGRRVDETSPICDARLPDGSRVNVIAPPLSIDGTTLTIRKFKKDKLTLDQLVQFGAISPEGAQILQIIGRVRCNVVISGGTGSGKTTLLNCLTRYIDTDERIITCEDSAELQLQQPHVVR

BbacteriumSG-6C VGRRVDESSPICDARLADGSRVNAIVPPLAIDGPALTIRKFKKDKLTLDQLVKFGAISPEGAQILQIIGRVRCNVLISGGTGSGKTTLLNCMTNYIDEDERIITCEDAAELQLQQPHVVR

Aclevelandensis VGRRVDESSPICDARLADGSRVNAIVPPLAIDGPALTIRKFKKDKLTLDQLVKFGAISPEGAQILQIIGRVRCNVLISGGTGSGKTTLLNCMTNYIDEDERIITCEDAAELQLQQPHVVR

Lsediminis RGRRIDESNPLVDTRLADGSRVNAVIPPLALKDPCLTIRKFPDERLSVKDLINFGSLTDEMAKFLARAVRSKKNIIISGGTGSGKTTLLNVLSGFIGQTERVVTIEDAAELQIYQEHVIS

Llitoralis RGRRIDESNPLVDTRLADGSRVNAVIPPLALKDPCLTIRKFPDERLTVKDLINFGSMTDEMAKFLARAVRSKKNIVISGGTGSGKTTLLNVLSGFIGQTERVVTIEDAAELQIYQEHVIS

Pcaeni RGRRIDESNPLVDTRLADGSRVNAVIPPLALKDPCLTIRKFPEDRLGVGDLVNFESLTPEMAKFLGRAVRARKNIIISGGTGSGKTTLLNVLSSFIGQTERVVTIEDAAELQLQQDHVVS

Bsediminis RGRRIDESNPLVDTRLADGSRVNAVIPPLALKDPCLTIRKFPEDRLGVGDLVGFKSMTPEMAKFLGRSVRARKNIVISGGTGSGKTTLLNVLSSFIGVTERVVTIEDAAELQMQQEHVVS

**:::: * * ** **:*::.: * :: . :::*:* : .: .::: ****:***: :* : : :*::* **:*** : : : :

Mxanthus LESRPPNLEGKGAITIRELVKNCLRMRPDRIVVGECRSGETLDMLQAMNTGHDGSLTTLHANTPRDAIARLETMVLMSGMDLPVKAIREQIASAVHMIVQQTRFSDGTRKICYITEVSGM

Bbacteriovorus LETRPASMEGSNAIHIRDLIKNALRMRPDRIIVGECRDGAALDMLQAMNTGHDGSMTTTHANSPRECVARLETLCMMSGMDLPMRAIREQIAGAVNLIVQISRLSDGSRKILSITEVAGM

Mmacrosporus LESRPPNLEGKGAITIRELVKNCLRMRPDRIVVGECRSGETLDMLQAMNTGHDGSLTTLHANTPRDAIARLETMVLMSGMDLPVKAIREQIASAVHMIVQQTRFSDGTRKICYITEVSGM

Mstipitatus LESRPPNLEGKGAITIRDLVKNCLRMRPDRIVVGECRSGETLDMLQAMNTGHDGSLTTLHANTPRDAIARLETMVLMSGMELPVKAIREQIASAVHMIVQQTRFSDGTRKICFVTEVSGM

Saurantiaca LESRPPNLEGKGAITIRDLVKNCLRMRPDRIVVGECRSGETLDMLQAMNTGHDGSLTTLHANTPRDAIARLETMVLMSGMELPVKAIREQIASAVHIIVQQTRFSDGTRKICFITEVAGM

Cfuscus LESRPPNLEGKGAITIRDLVKNCLRMRPDRIVVGECRAGETLDMLQAMNTGHDGSLTTLHANTPRDAIARLETMVLMSGMELPVKAIREQIASAVHLIVQQTRFSDGTRKICFITEIAGM

Dretbaense LETRPPNIEGKGEVTARELVRNSLRMRPDRIIVGEVRGSESFDMLQAMNTGHDGSLTTIHANTPRDALMRIESMVSMANLDIPIEFMRRFIASAIHIIIQVSRYSDGTRKVNSIQEITGM

Paespoeensis LETRPANIEGRGEIGQRELVKNCLRMRPDRIILGEVRASEALDMLQAMNTGHDGSLTTVHANTPRDALMRLETMVSMAGLNLSPISMKRYISSAIEVIIQATRLVDGTRKVISIQEVTGM

Ppiezophilus LETRPANIEGKGEIDQRELVKNCLRMRPDRIIVGECRASEALDMLQAMNTGHDGSLTTIHANTPRDALMRLETMVSMAGLNLSPMSMKRYISSAIDVIIQATRMVDGTRKVISIQEVSGM

Dtoluolica METRPANIEGKGEIIQRDLLKNSLRMRPDRIIVGEVRGSESFDMLQAMNTGHDGSLTTIHANSARDALMRLETMVAMANFDIPSEFIRRFISSAIHIVIQVSRLSDGKRKVVSLQEITGM

OsymbiontRs1 LESRPANLEGKGAIPIRDLVKNCLRMRPDRIVVGECRGGEALDMLTAMNTGHDGSLTTVHANTPRDVVSRLEVMVMMAGMDLPARAIREQIASAVHVIVQQSRLADGSRKITHISEITGM

Dsulfexigens LEARPANMEGKGAIIIRDLVKNCLRMRPDRIIVGECRGGETLDMLQAMNTGHDGSLTTVHANSPRDLVSRLEVMVMMAGMDLPEKAIREQIASAVHVVVQQSRFSDGTRKITHITEITGM

Samazonensis LEARPPNQEGKGAIEIRDLVKNCLRMRPDRVVIGECRGGEALDMLQAMNTGHDGSLTTAHSNSPRDCISRLEVMVMMSGMDLPIQAIREQIASAVNIIVQQSRFSDGSRRITSICEVTGM

Swoodyi LEARPPNQEGKGAIEIRDLVKNCLRMRPDRVVIGECRGGEALDMLQAMNTGHDGSLTTAHSNSPRDCISRLEVMVMMAGMDLPVSAIREQITSAVNIIVQQSRFSDGSRRITSICEITGL

Bgladioli LEARPPNMEGKGAVPIRDLVKNCLRMRPDRIVVGECRGGEALDMLQAMNTGHDGSLTTAHANSPRDCIARLEVMTLMAGLDLPVQAIREQICSAVDIIVQQTRFSCGSRRVTHVTEVSGM

Bsp.CCGE1001 LEARPANMEGKGAVNIRDLVKNCLRMRPDRIVIGECRGGEALDMLQAMNTGHDGSLTTAHANTPRDCIARLEVMTLMAGLDLPVHAIREQICSAVDIIVQQSRFSCGSRRVTHITEVSGM

Sloihica LEARPPNQEGKGAIEIRDLVKNCLRMRPDRVVIGECRGGEALDMLQAMNTGHDGSLTTAHSNSPRDCISRLEVMVMMAGMDLPVTAIREQITSAVNIIVQQSRFCDGSRRVTSICEVTGI

Aferrivorans LEARPANMEGKGQIPIRELVRNALRMRPDRIVVGECRGGEALDMLQAMNTGHDGSLTTAHANTARDMLSRLEVMVLMAGMDLPLTAIREQIASAVDIIVQITRFSCGSRKVTSICEVTGT

Pxenovorans LEARPANMEGKGAIHIRDLVKNCLRMRPDRIVIGECRGGEALDMLQAMNTGHDGSLTTAHANTPRDCIARLEVMTLMAGLDLPVQAIREQICSAVDLIVQQSRFSCGSRRVTHVTEVSGM

Csp.GA3-3 LEARPPNMEGKGAVTIRDLVKNCLRMRPDRIVVGECRGGEALDMLQAMNTGHDGSLTTAHANSPRDCLSRLEVMTLMAGLDLPVQAIREQVCSAVDVIVQQTRFSCGSRRVTHITEVSGM

Tsp.28 LEARPPNAEGRGQVAIRDLVKNALRMRPDRIVVGECRGGEALDMLQAMNTGHDGSLTTLHANSPRDALARLETLVLMAGMDLPLSAIREQIASAIDIVVQQARLPDGRRVVTGIVEVAGT

Cnecator LEARPPNMEGKGAVTIRDLVKNCLRMRPDRIVVGECRGGEALDMLQAMNTGHDGSLTTAHANSPRDCLSRLEVMTLMAGLDLPVQAIREQVCSAVDVIVQQTRFSCGSRRVTHITEVSGM

Tterpenica LEARPANAEGRGQIAIRDLVKNALRMRPDRIVVGECRGGEALDMLQAMNTGHDGSLTTLHANSPRDALARLETLVLMAGMDLPLTAIREQIAAAIDLVIQQARLPDGRRVITAIVEVAGT

Acaldus MEARPANVEGKGAIPIRELVRNALRMRPDRIVVGECRGGEALDMLQAMNTGHDGSLTTAHANSPRDMLSRMEVMVMMAGMELPLAAIREQIASAIDIIVQITRFSCGSRKLTAIVEVTGT

Veiseniae LESRPANVEGKGGVAIRELVRNTLRMRPDRIVVGECRGAEALDMLQAMNTGHEGSLTTLHANSPRDGLARLETMVLMAGMELPLAAIREQIASAVDILVQQTRFSCGTRMVTHITEISGM

Hneapolitanus MEARPANLEGKGQIAIRDLVRNSLRMRPDRIVVGECRGGEALDMLQAMNTGHEGSLTTAHANTPRDALSRLEVMVLMSSMDLPISVVREQIASAIDLIIHQRRFPCGSRKITHICEVTGL

Phospita LETRPANLEGKGAVTIRELVRNCLRMRPDRIVVGECRGGEALDMLQAMNTGHDGSLTTAHANSPRDCIARLEVMTLMAGLDLPVQAIREQVCAAVDIIVQQTRFSCGSRRVTHITEVSGI

Dacidovorans LESRPANVEGKGGVPIRELVRNTLRMRPDRIVVGECRGAEALDMLQAMNTGHEGSLTTLHANTPRDGLARLETMVLMAGMELPLAAIREQIASAVDIIVQQTRFACGTRLVTHICEVSGM

Taminoaromatica LEARPSNLEGRGAISIRDLVRNALRMRPDRIVVGECRGGEALDMLQAMNTGHEGSMTTLHANSPRDALARLETLVLMAGMDLPLAAIREQIASAVDIIVQQTRFACGARRLTSITELTGM

Smaltophilia LEARPPNMEGKGHISIRDLVRNALRMRPDRIVVGECRGGEALDMLQAMNTGHEGSLTTAHANNPREALSRLEVMVMMSGMELPMTVVREQISSAVDLIVHQKRFPCGSRKVSHITELTGM

Pphymatum LETRPANLEGKGAVTIRELVRNCLRMRPDRIVVGECRGGEALDMLQAMNTGHDGSLTTAHANSPRDCIARLEVMTLMAGLDLPVQAIREQVCAAVDIIVQQTRFSCGSRRVTHITEVSGI

Ahongdengensis LEARPANQEGRGAINIRDLVRNCLRMRPDRIVVGECRGGEALDMLQAMNTGHDGSLTTIHSNSPRDCISRLEVLVLMSGMDLPVHAIREQIASAVDIIVQQTRFACGSRKVTSIAEVTGL

Rspathiphylli MEARPANLEGKGLITIRDLVRNSLRMRPDRIVVGECRGGEALDMLQAMNTGHEGSLTTAHANSPRDALSRLEVMVMMSGMDLPMTVVREQIASAIDLIIHQRRFPCGSRKITHISEITGI

Psp.T7-7 LESRPANLEGRGNIAIRDLVRNALRMRPDRIVVGECRGGEAFDMLAAMNTGHEGSLTTLHANSPRDALARLETMILMAGMDLPLSAVREHIAASIHFIVQQARLNNGRRLITSIVEIAGM

Vparadoxus LESRPPNIEGKGAIAIRDLVRNSLRMRPDRIVVGECRGGEALDMLQAMNTGHDGSLTTLHSNSPRDAMSRLEVLVTMAGMNIPVAAIREQIASAVDLIVQLTRFPCGSRKVTQITEITGF

Amarplatensis LEARPANQEGRGQIDIRELVRNALRMRPDRIVVGECRGAEAFDMLTAMDTGHEGSLTTLHANSPRDALGRLESMILMAGLDLPLSAVREHIAASVDLVVQQARLADGRRVVMSIVEVAGM

Aarsenitoxydans LEARPANQEGRGRIDIRELVRNALRMRPDRIVVGECRGAEAFDMLTAMNTGHEGSLTTLHANSPRDALARLESMILMAGLDLPLSAVREHIAASVDIVVQQARLTDGRRVVTSIVEVSGM

Axylosoxidans LEARPPNQEGRGRVDIRELVRNALRMRPDRIVVGECRGAEAFDMLTAMNSGHEGSLTTLHANSSRDALARLESMVLMAGLDLPLTAVREQIAASIDIIVQLARQPDGRRVVLSIAEVTGM

Dvulgaris LETRPANIEGHGQVTARDLVKNCLRMRPDRIIVGEVRSGEVLDMLQAMNTGHDGSLTTIHANTPRDCLMRLETMVAMAGLNIGTLSLKRYIASAVDVIIQVSRLADGSRKMTSLSEITGM

Bexovorus LETRPPSMEGSNAVTIRDLIRNALRMRPDRIVVGETRDGAALDMLQAMNTGHDGSMTTTHANSPRECLARLETLVMMSGLELPVRAIREQIAGAVDLIVQIVRLSDGSRKIISITEVVGM

Delongatus LESRPPNIEGKGEVTLTDLVKNSLRMRPDRIIVGEARGGEVMDMLQAMNTGHPGSMSTIHANTPKDALSRMEVMAGMGTALFSERALRGLIASAIHLIVQLARLPDGVRRVVSISEIHGI

Hmarinus LETRPPSLEGDGEIDIRCLVKQTLRMRPERIVVGECRGGETLDMLQAMGTGHDGSMTTVHSNNPRECIGRLETLVQYAGAGLSPKAIREMIANSVHMIIQQKRLDDGSRKMTHISEIGGI

Bstolpii LETRPPSLEGDGAIDIRCLVKQTLRMRPDRIVVGECRGGETLDMLQAMGTGHDGSMTTVHSNNPRECIGRLETLVQYAGTSISPRAIKEMIANAVHMIIQQSRLDDGSRRVMYITEIAGM

Dphenolica METRPANIEGKGEIIQRDLLKNSLRMRPDRIIVGEVRGSESFDMLQAMNTGHDGSLTTIHANSARDALMRLETMVAMANFDIPSEFIRRFISSAIHIVIQVSRLSDGKRKVVSLQEITGM

Dcetonica LESRPPNIEGRGEVTLTDLVKNSLRMRPDRIIVGEARGAEVMDMLQAMNTGHPGSMSTIHANTPKDALSRMEVMAGMGTALFSERALRGLIASAIHLIVQLARLPDGSRRVMSISEVHGM

Dmultivorans LETRPPNIEGKGEVTQRDLVKNALRMRPDRIVLGEVRSSEAFDMLQAMNTGHEGSLTTIHANTPRDALMRLESMVAMANFDIPSEFMRRFISSAINVIIQLTRFSDGTRRLTNFQEITGM

Cvibrioides LETRPPNLEGQGSVTMRDLVKNCLRMRPERIIVGEVRGPEAFDLLQAMNTGHDGSMGTLHANSPREAISRVESMITMGGYGLPSKTIKEMIVGSVDVIIQAARLRDGSRRITHVTEVVGL

Atumefaciens LETRPPNIEGEGEITMRDLVKNCLRMRPERIIVGEVRGPEVFDLLQAMNTGHDGSMGTIHANTPRECLSRMESMIAMGGFSLPAKTVREIISSSVDVIIQAARLRDGSRRITQVTEVLGM

Aexcentricus LETRPPNLEGQGAITMRDLVKNCLRMRPERIIVGEVRGPEAFDLLQAMNTGHDGSMGTLHANSPREAISRLESMITMGGYGLPSRTIREMIVGSVDIIVQAARLRDGSRRITHITEVLGL

Bdiminuta LETRPPNLEGQGQITMRDLVKNCLRMRPERIIVGEVRGPEAFDLLQAMNTGHDGSMGTLHANSPREAISRMESMITMGGYGLPSKTIREMIVGSIDVIVQAARLRDGSRRITHITEVVGL

Mmaris LETRPPNLEGSGMVTMRDLVKNCLRMRPERIIVGEVRGPEAFDLLQAMNTGHDGSMGTLHANSPREAMSRIESMITMGGYNLPAKTIREMIVGSIDVIVQAARLRDGSRKITHITEVVGM

Plavamentivorans LETRPPNLEGQGQVTMTELVRNCLRMRPERIIVGEVRGPEAFDLLQAMNTGHDGSMGTLHANSPREALSRLESMITMGGYALPSKTIREMICGSIDVVIQAARLRDGSRRITHITEVLGT

Nhamburgensis LETRPPNIEGEGQVSMRELVRNCLRMRPERIIVGEVRGPEAFDLLQAMNTGHDGSMGTLHANNPREALSRCESMITMGGFALPSRTIREMICASIDIIIQAARLRDGSRRITHITEVMGM

Amanganoxydans LETRPPNIEGEGEITMRDLVKNCLRMRPERIIVGEVRGPEVFDLLQAMNTGHDGSMGTIHANSPRECLNRMESMIAMGGFSLPSRTVKEIIVGSIDVIVQAARLRDGSRRITHITEVLGM

BbacteriumSG-6C LETRPPNIEGEGQITMRDLVKNCLRMRPERIIVGEVRGPEAFDLLQAMNTGHDGSMGTLHANNPREALSRVESMITMGGYSLPSRTIREMICASIDVIVQAARLRDGSRRITHITEIMGM

Aclevelandensis LETRPPNIEGEGQITMRDLVKNCLRMRPERIIVGEVRGPEAFDLLQAMNTGHDGSMGTLHANNPREALSRVESMITMGGYSLPSRTIREMICASIDVIVQAARLRDGSRRITHITEIMGM

Lsediminis LETKPPNLEGKGEIGIRELVKNALRMRPDRIVVGECRGGEALDMLQAMNTGHDGSMTTVHSNSPTEAIARLETLVLMSGMELPTRAIREQIAGSVDVIIQQSRFMDGSRRITYITEVVGI

Llitoralis LETKPPNLEGKGEIGIRELVKNALRMRPDRIVVGECRGGEALDMLQAMNTGHDGSMTTVHSNSPTEAIARLETLVLMSGMELPTRAIREQIAGSVDVIIQQSRFMDGSRRITYITEVLGI

Pcaeni LETKPPNVEGKGEVSIRDLVKNALRMRPDRIVVGECRGGEALDMLQAMNTGHEGSMTTVHSNSPREAIARLETLVLMSGMELPTRAIRQQIANSVDVIVQQSRFSDGSRRVSYITEVIGI

Bsediminis LETKPPNLEGKGEVSIRDLVKNALRMRPDRIVVGECRGGEALDMLQAMNTGHDGSMTTVHSNSPTEAISRLETLVLMSGMELPSRAIRQQIANSIDLIVQQSRFSDGSRRISYITEVCGI

:*::*.. ** . : *::: *****:*:::** * :*:* **.:** **: * *:*.. : : * * : . : :: : ::..::: * * * : . *: *

Mxanthus -EVDIVTLQDIFYFKQDGFTEDH----KVRGRYVASGF-VPK-FLIKAGGMYYPLMWLNDQVKKRHLLISRALPYSLDLLTLSVEAGLDFTAALAKVVEK----GKPGPLREELQLVLKQ

Bbacteriovorus -QGDVVTLAEIFRFKETGYDKNR----KIQGVFQATGT-IPS-FIKTSGGFYLPQIHARGEKKRRELSVRADLPFFIDLLALSVEAGLDFFSAIQKIVDKA--NGTDSVLAEEFSIVLKD

Mmacrosporus -EVDIVTLQDIFYFKQDGFTEDH----KVRGRYVASGF-VPK-FLIKAGGMYYPLMWLNDQVKKRHLLISRALPYSLDLLTLSVEAGLDFTAALAKVVEK----GKPGPLREELQLVLKQ

Mstipitatus -EVDIVTLQDIFYYKQDGFTEDH----KVRGRFVASGF-VPK-FLIKAGGMYYPLLWLNDQVKKRHLLISRALPYNLDLLTLSVEAGLDFTAALAKVVEK----GKAGPLREELQLVLKQ

Saurantiaca -EVDIVTLQDIFYYKQDGFTEDQ----KVRGRFVASGF-VPK-FLIKAGGLYYPAIWVNDQVKRRHLAISRALPYNLDLLTLSVEAGLDFTAALSKVVEK----GKSGPLKEEMSIVLKQ

Cfuscus -EVDIVTLQDIFYYKQEGFTDEG----KVRGRYVASGF-VPK-FLIRAGGMYYPLIWVNDQVKKRHLLISRALPYNLDLLTLSVEAGLDFTGALAKVVEK----GKAGPLREELQIVLKQ

Dretbaense -EGNVITTQEIFSFNPTGVDENG----KVKGYFRFNGV-RPQ-FFLKAGGFYVPDLWLTNKRQKRRQTILKGFPDALDLMVVCVEAGMGLDAAISRVAKES--KSNNATLSEELHFFTLE

Paespoeensis -EGEMITMQEIFAFEQTNVSAEG----KVEGYFTARGI-RPR-FLVQAGGVYGPEVWLRRRTTKRRLAVANELPDALDLLVVCVESGMGLDQALDRVCHEM--RTSGPVISAEFKLLTLE

Ppiezophilus -EGEMITMQEIFSFEQTNVSKQG----KVEGYFTARGI-RPK-FLIQAGGIYGPEFWLKKRIANRQLAVANELPDALDLLVVCVESGMGLDQAIERVQREL--RNSGPIISLELKILTLE

Dtoluolica -EGNMITMQEIFSFKQTRVDEKG----DVKGFFRFNGI-RPK-FFLRAGGFYLPEIWLKQKTDKRKEILFKGLPDALDLLLVCVEAGMGLDSAMNRVGQEL--RLTYPDLSREFSLMNLE

OsymbiontRs1 -EGDTVQLNDIFVYRQEGFDEEG----KVVGQYMPTGQ-VPH-FLQHTGGFMFPSVWLADTRKRREMQVIRALPIYLDFTTMAVEAGLNLTGAMQQALNK----GPKGALRNEFSIVMRD

Dsulfexigens -ETGVVQMQNIFIYKQNGLDKDG----NVIGEYKATGR-VPE-FLKRNGGFFYPDIWLRDIRKKQVDAVLRTLPAYLDFITMAVEAGLNFSGAIAQARTK----GPAGPLVIEFGIVLRD

Samazonensis -EGSTVQLSEIFKFQQTGFSEQG----KVMGFYTATGT-IPE-FLQVSGGYFLPMLTLNDLRRTRQEQIVKVLPVYLDYLTMAIEAGLNMSAAIAQAVER----GPDGPLKIEFEKVIRD

Swoodyi -EGSIVQLSEIFKFEQTGFNSEG----KVQGYYTATGT-MPE-FLQISGGYILPVITLNDLRKKRQALIVKSLPVYLDYLTMSIEAGLNMTGAISQAVER----GPSGPLRIEFEKVIRD

Bgladioli -ESGVIQLQDVFVFRQDGFGEDG----RVQGVFHPTSY-VPD-FLRLTHGYSYPRIWVRDVRRARVVAIRKQLPVYLDFLTLAVEAGLNINGAIQKAVEK----GPDGALRSEFEHVLRD

Bsp.CCGE1001 -ESGVITLQDVFVFKEEGFSEQG----KIQGKFVPTAY-VPD-FLRLTSGYFYPRIWTRDVRRRRVAQILKHLSIYLDFLTLAVEAGLNINGAIQKAVEK----GPAGPFRWELEHVLRD

Sloihica -EGTVVQLSEIFKFQQTGFDEQG----KVQGYYTATGT-LPE-FLQVSGGYFLPVMTLNDLRKKRQGLIVKSLPVYLDYLTMSIEAGLNMTGALSQAVER----GPAGPLRIEFEKVIRD

Aferrivorans -ESGTIQLQELFTYKQRGYDAEG----RVRGIFRATGA-VPE-FLQRAGGGFYPDLWLVEQAKRRGRLILKELPVYLDFITLMIEGGLNLTSAMQVAVDK----GPLGPLRNEFSIMIRD

Pxenovorans -ESGVITLQDVFVFKEEGFSEHG----KIQGKFVPTAY-VPD-FLRLTSGYFYPRIWTRDVRRRRVAQILRHLPIYLDFLTLAVEAGLNINGAIQKAVEK----GPEGPLRRELEHVLRD

Csp.GA3-3 -ETGVVQLQDVFMFREEGYGADG----KVKGRYVATSY-IPD-FLRLTGGFFYPRIWLRDVRKRNLLQVTRHLPVFLDFLTLSVEAGLNINGALLQAVEK----GPEGPLRREFEHVLRD

Tsp.28 -ESGRIQIQELFRYDQLGRDGDG----RVQGRFCACNA-VPA-FLRLAGGAFLPLAWLRDRIQRRRRQTFKGLPFMLDLITLCVESGLNLNSAIAQAVAK----GPAGPLRDEFARLLRD

Cnecator -ETGVVQLQDVFMFREEGYGADG----KVKGRYVATSY-IPD-FLRLTGGFFYPRIWLRDVRKRNLLQVTRHLPVFLDFLTLSVEAGLNINGALLQAVEK----GPEGPLRREFEHVLRD

Tterpenica -ESGRIQMQELFRHEQRGRDAEG----RVRSRFTGCNA-IPA-FLRLAGGYVFPLVWLRDRIALRRRLTFKALPFMLDLITLCVESGLNLNSAIAQAVAK----GPTGPLRDEFARLMRD

Acaldus -ESGVIQLQELFYFKQQGFGPDG----KVRGTFRASGS-IPE-FIQKAGTSFYPDLWLVEQAKRRQKRILKDLPVYLDFITLMVEGGLNLTGAIQIAVDK----GPEGPLRNEFRIILRD

Veiseniae -ESGKIQLQDLFGFVNRGYAMQADGLSKVQGHFSGCDT-VPH-FLRQAGGWFYPAIWLKDRIALRRRELLRMMPFFLDIITLCVEAGLNMHGAIAQAVAK----GPQGTVREEFQRVLRD

Hneapolitanus -ESGTIQTQDIFVFRGREQGGAG---GKVRGSFSATGA-VPE-FLQTAGGWLYPSLWLTERRKHRQRLVIKDLPVFLDFIVMAVEAGLNITGGIEQATLK----GPKGPLAQEFRRLLRD

Phospita -ESGVIQLQDVFVFREAGLDDNG----KIRGQFVPTSY-IPD-FLRLTSGYVYPRIWMRDVRRRHVAMVLRQLPVYLDFLTLAVEAGLNINGALQKAVEK----GPDGPLRREFEHVLRD

Dacidovorans -ESGKIQLQELFRFANKGYAAQANGLAKVQGLFTGCDV-VPH-FLRQAGGWTYPAIWLRDRIAVRRRELLRMLPFFLDIITLCVEAGLNMQGAMTQAVAK----GPQGLVRDEFQRVLRD

Taminoaromatica -EGGRIQLQELFRFERR--TADSPTADPSGGHFTGCDA-VPG-FLRLAGGTLIAWSWLDDRIRCRRRLMLKQLPFVLDLITLCVEAGLNLTGALQQAAAK----GPAGPLGEELHRVLRD

Smaltophilia -ESGTIQLQDVFLFKARSTAGRD---GKVVGEFVATGA-VPE-FLQSAGGWMYPTLWLGERRKARSKHVIRDLPIFLDFITMAVEAGLNITGAIEQSVDK----GPPGPLSQEFSRMLRD

Pphymatum -ESGVIQLQDIFVFREAGLGENG----KIRGQFMPTSY-IPD-FLRLTSGYFYPRIWMRDVRRRYVATVLRQLPIYLDFLTLAVEAGLNVNGAIQKAVEK----GPDGPLRREFEHVLRD

Ahongdengensis -EGGVIQLSEIYRFQQKGYDQEG----RVRGDFTATGA-IPD-FLAQSGGFALPYLNVRERRKKRRVEILRQLPVFLDYLAMSIQAGANFTGAIRHSVEK----GPTGPMKAELNKVMRD

Rspathiphylli -ESGTIQAQDLFTFRARDQGGPG---GKVRGEYAATGA-VPE-FLQTAGGWFYPSLWLGERRSKRRRLVIKDLPVYLDFITMAVEAGLNITGGIEQATQK----GPAGPLRQEFTRLLRD

Psp.T7-7 -ESGRIQTQELFRYLRSP-----------TPAFVGCGV-VPECFVLTAGGMLWPRYWLHGLARQRQFQMLREFPFLLDMTTLCVEAGLNLHGALQQAAQH----GPDGPLRGELRHALAD

Vparadoxus -ADGMIQMQDVYVFRKTGVDAEF----KTVGHFAAAGT-VPD-FLQLAGGWYYPVMWMRDRRKKREKNILRTLPGYLDMITLCCQAGLSLTGAIAQAVQK----GPGGALGIEFDRMMRE

Amarplatensis -ESGRIQLLELFRYDRA-------------AGFQGCGA-LPG-FIEQAAAGVAPGLWLRGLGEKRRRSIERDLPFVFDMMTLCVEAGLSVQGALQMAAQS----GPRGALRDGLADALAE

Aarsenitoxydans -ESGRIQLQDLFRHDRN-------------RGFVGCGG-LPS-FIEQAASGAAPGLWLRRLGDQRRRNIERDLPFVFDMMTLCVEAGLSVQGALQLAAQS----GPPGVLRDALADALAE

Axylosoxidans -ESGRIQLQDLFRHERA-------------GGFSGCGT-IPS-F-MRAGAALLPFAWLRAQGEARRRGIERGLPFMLDMMTLCVEAGLGAHAALQAAAAN----GPSGPLRDALAGALAQ

Dvulgaris -EGDAITMQDIFTFEQTGVDENG----KVQGRFRSGGI-RPR-FLVRAGGLYAPNVWLSRRIKARRLDIINSLPDALDLLVVCVEAGMGLDQAFSRVAREM--AATNPVLAEELNLVILE

Bexovorus -QGDVITLAEIFKFKETGYDKNR----RVLGQFQSTGT-VPT-FIKVGGGFFMPQMHCKGTKKQRELSVRADMPFFIDLLALSVEAGLDFFGAIQKIVDKV--ADKDSVLAEEFKIVLKD

Delongatus -RDGTIMVEPLVVFRQENVDEQG----RIVGSFQGTPA-VPF-FLSYAGGYFFPTILLNMRIRSRQAQIFRELPDALDLVLICLEAGLSFDAALFRVSREL--ANVSPVLAGEFSQYFLE

Hmarinus -QGDVVTLQDIFLFQQRDIDKNG----KIIGEFQATGF-IPK-FLASAGGFVYPDIWIKGRIEMRQKEVIMAMPFCVDMLALSVEAGLDFVAAMTKVVEKA----KKTALSEEFETMIKE

Bstolpii -QGDTVILQDIFLFIQKEIDKNG----KILGEFQATGF-IPK-FLAAAGGFFYPDFWIKGKIQQRQKDIISAMPFCVDMLALSVEAGLDFIAAMTKVIEKA----KANPLTEEFEILIKE

Dphenolica -EGNMITMQEIFSFKQTKVDEKG----DVKGFFRFNGI-RPK-FFLRAGGFYLPEIWLKQKTDKRKEILFKGLPDALDLLLVCVEAGMGLDSAMNRVGQEL--RLTYPDLSREFSLMNLE

Dcetonica -ADETVAVVPLVAFVQEGVDDRG----RVLGCFKGTGK-ASL-FLTHGGGYYLPAAWLRIRIRSRQKEIFRELPDALDLVLICLEAGLNFDLSLYRVSREL--KRVSPVMSEEFGQYFLE

Dmultivorans -EGNVITLQEIFSFQQTGLDDKG----GVRGCFRFHGI-RPR-F-----GLYLPEIWIDLKITKRKDLIAKGLPDALDLMVVCVEAGMDLNAAMNKVAEEI--HLTCPPLSEELRMMSLE

Cvibrioides -EGDVIVTQDLFVYEITGEDENG----KVLGKHRSTGIARPR-FLAQAGGYYAPNVYISNVAQKRRESIVAAFPDALDLLLICVESGMSIEAAIQKVGAEV--GSSSMELAEELSLLTAE

Atumefaciens -EGDVIITQDLMRYEMDGEDANG----RLIGRHVSTGISKPY-FLRAAGGFYAPNIYVSNRMTKRQASIKRAWPDALDLMLICIESGVSMEAAMRRVAEEM--GEQSPELAEEMMLTTAE

Aexcentricus -EGDVIITQDLFLYEIEGENKEG----KIIGRHRSTGIGRPR-FLAMAGGYYAPNIYISNAASKRRASIVTAFPDALDLLLICVEAGMSIEAALQKVSQEI--GSASIELAEELSLLVAE

Bdiminuta -EGDVIVTQDLFVYDITGEDAHG----KITGRHRSTGIARPR-FMIQAGGFYAPNIYLKNMIDKRRASIMAAFPDSLDLLLICVESGMSIEAAIQKVSQEV--GGQSIELAEELSLLSAE

Mmaris -EGEVIVTQDLFLYEIKGEDEHG----KIVGQHASTGIARPK-FLAQAGGYYAPNLYLSNLAAKRQQSIMGAFPDGLDLLLICVESGMSIEAAFHKVSSEV--GSQSIELAEELSLTTAE

Plavamentivorans -EGDVIITQDLFVYDIEGEDETG----RIIGKHKSTGIARPS-FLRQAGGFYLPNLFVENVISRRQDSIRKAFPDALDLMLICVESGMSIEAAFQKVAAEI--GAQSVELAEELGLTTAE

Nhamburgensis -EGDTIITQDIFIYDLLGEDANG----NVVGRHRSTGIGRPR-FLVMAGGLQAPMLFLKNAITKRQISIKRAFPDALDLLLICIESGMSIEVAFRKVSTEI--GSQSVALAEEFTLTTAE

Amanganoxydans -EGDVITTQDLFVYDMTGEDAQG----RIIGRHRSTGIGRPA-FLRNAGGFVAPIFFVKNRATKRRLSISRAWPDALDLLLICVESGNSIEAAFRRVAEEI--GIQSIPLAEELVLVCAE

BbacteriumSG-6C -EGDTIITQDIFLYDMMGEDLNG----NILGRHRSTGIGRPR-FLVMAGGMQAPMLFLKNAITKRQLSIKRAFPDALDLLLICIESGMSIEAAFRRVGQEI--GSQSIPLAEEFSLTTAE

Aclevelandensis -EGDTIITQDIFLYDMMGEDLNG----NILGRHRSTGIGRPR-FLVMAGGMQAPMLFLKNAITKRQLSIKRAFPDALDLLLICIESGMSIEAAFRRVGQEI--GSQSIPLAEEFSLTTAE

Lsediminis DDDGFVKLEDIYRYRQKGVNEKG----KVYGYHLATGY-LPS-FLMRSGALGFPWLSLSGAISSRLIEIDRRLPYTVDLLVLSMRAGLDFMSALDRVVARGQIQNPDDPMIQELGVVLQE

Llitoralis DDDGFVKLEDIYRYRQKGINEKG----KVYGYHMATGY-LPS-FLMRSGSLGFPWLSLSGAISSRLIEIDRRLPYTVDLLVLSMRAGLDFMTALDRVVVRGQLQNPDDPMIQELGVVLQE

Pcaeni NDDGYVELEDIYRYKQTHVDENK----KVHGYHMATGY-LPS-FLPRSGGLYFPFLNLRGAITERLIEIDRRLPYTIDLLVLSMRAGLDFMTALDRVVTRGQEQNPDDPMIQELGVVLQE

Bsediminis DEDEHIRLEDIYRYRQTHVDQDM----KVHGYHLATGY-LPS-FLPRSGSLYFPFLSLSGAIANRLIEIDRRLPYTIDLMVLSMRAGLDFMTALDRVVTRGQQQNPDDPMIQEFGVVLQE

: : . . . * . . .* : ..* . .: . : :

Mxanthus LKMGKTREEGLKSMIVRVDLPPLTTFVTALIQADKMGTSLGKVLRIQSTQMRIDRTQRAEKLAGEAPVKMLFPLIACIFPTVFMVLFGPPMLMVKHYRKRRIKKFNVQLVDALQAMANAF

Bbacteriovorus IKIGSSKAQALKEMAERLDMNEITSFVAVLVDAESTGASISQVLKDQSVQMRLERFVRAEKAGAQASQTMLIPLMLFILPAVFIIVFGPPLLLVRMIYEQRCTKFVDQMVDGLTIMANGI

Mmacrosporus LKMGKTREEALKSMIVRVDLPPLTTFVTALIQADKMGTSLGKVLRIQSTQMRIDRTQRAEKLAGEAPVKMLFPLIACIFPTVFMVLFGPPMLMVKHYRKRRIKKFNVQLVDALQAMANAF

Mstipitatus LKMGKTREEGLKSMIVRVDLPSLTTFVTALIQADKMGTSLGKVLRIQSTQMRIDRTQRAEKLAGEAPVKMLFPLIACIFPTVFMVLFGPPMLLVKHYRKRRIKKFNIQLVDALQAMANAF

Saurantiaca LKMGKTREEALKAMILRVDLPSLTTFVTALIQADKMGTSLGKVLRIQSTQMRIDRTQRAEKLAGEAPVKMLFPLIACIFPTVFMVLFGPPMLLVKYYRKRRIKKFNVQLVDALQAMANAF

Cfuscus LKMGKTREEGLKAMIARVDLPSLTTFVTALIQADKMGTSLGKVLRIQSTQLRIDRTQRAEKLAGEAPVKMLFPLIACIFPTVFMVLFGPPIILVKHYRKRRIKKFNVQLVDALQAMANAF

Dretbaense TRAGMPRKDALKNLAMRTDLQQMQNLTTLLIQTEKFGTSVSQALKVFSESMRTERFQRAEEKAAKIPVKLLFPLIMFIFPSLFVAILGPPFFYLKRKKLKRMRKFMAQLPEGLDLLARSL

Paespoeensis LRAGKGRGDALRGLSERVGLDDLNSLTSLLVQADIFGISVGRTLRVYSDAMRTKRSQRAEEKAAKLPVLLLLPLIGFILPALFIAIMGPPVWHLRRLKARRMGRFQAQLPEALDLMSRAL

Ppiezophilus LRAGKGRADALRSLSERVGQEDLSSLTSLLVQADIFGISVGRTLRVYSDAMRTKRSQRAEEKAAKLPVLLLLPLITFILPALFIAIMGPPILYLQKLRTSRMDRFQTQLPEALDLMSRAL

Dtoluolica MRAGKMRQDALRNLARRTGIDEMASLVTLLIQTDKFGTSVGSALKVFSDSFRTKRFQKAEEIAAKLPVNILFPLILFIFPSLFVVILGPPCLYLKYLKDKRIQKFKQQLHEALDLIARAL

OsymbiontRs1 MRSGVTRAEALKRMDARLRVDDISRFVNAMIQAEKMGSSMAKTLRIQSEQRRTERFQRAEKKAMEAPVKLVFPLIVFIFPVTFMILGFPPLWVYKRMRKNRLKKFEQQLPDVLVMISGAL

Dsulfexigens MRSGIPRTRALKRMAKRLDIHEISSFVNAVVQAEKMGSSMAGVLRIQAEQRRSERFQRAEKKAMEAPVKLIGPLVIFIFPTTFIVLAFPPYLILKEMIKKRLKKFEQQLPDALVMVAGSI

Samazonensis MRAGISRAQAFRNMADRVQISELNSLVSALAQAERTGASLGYTLRIQADQRRVERFQRAEKKAMEAPIKLMFPLIMFIFPVTFLILLFPPRYLYRYLHQRRRRRFVHQLPDALNMIAASM

Swoodyi MRAGMSRAQSFRNMAERVQITEINSLVSALAQAEKTGASLGQTLRVQSDQRRIERFQRAEKKALEAPVKLVFPLIVFIFPVTFMILAFPPRFAYKFLHKRRRRKFVHQLPDALNMIASSM

Bgladioli LKSGLNRADALRRFDERQDIKEITNFVGAVIQAERMGSGLAGTLRFQSEQRRAERFQRAEKLAMEAPVKLVFPLMMFIFPVTFVVLGFPPRRVLAIMRKRRIAMLERQLPDVLLMTAGAL

Bsp.CCGE1001 LKSGLNRTEALRRLDDRLRIKEVTNFVGAVVQAERMGAGLAKSLRFQSEQRRSERFQRAEKQAMEAPVKLVFPLLVFIFPITFIVLGFPPRKYFERMRKKRIEVIEQQLPDALLMMSGAL

Sloihica MRAGMSRAQAFRNMADRVQVTEVNSLVSALAQAERTGASLGQTLRIQSDQRRIERFQRAEKKALEAPVKLVFPLIVFIFPVTFMILAFPPRFAYQFLHKRRRRKFVHQLPDALNMIASSM

Aferrivorans LRAGTPKMDIFRRFGERVPLPEVRSLVSALIQSEERGGDLGPVLRAQAEQRREERFLRAEKLALEAPVKLLAPLVVFIFPITFLMLAFVPRFVLRWLQKRRLDRLHAQLPDALMMLASGL

Pxenovorans LKSGLNRTEALRRLDDRLRIKEVTNLVGTVVQAERMGSGLAKSLRFQSEQRRSERFQRAEKQAMEAPVKLVFPLLFFIFPITFIVLGFPPKKYLKRMRKKRIEALEKQLPDALLMMSGAL

Csp.GA3-3 LKSGLSRADALRRMDDRLRVKEVTNFVGAVIQAERMGSGLAATLRFQSEQRRTERFQRAEKQAMEAPVKLIFPLVVFIFPVTFIVLGFPPRALVKKMQRKRLMAIEDQMPDALLMMSSAL

Tsp.28 VRAGKARSEALRELASRLDLPAVSNFVTTLIQAEATGMSLGPILRAQAEQRRTERFAQAEKLAMQAPVKLLFPLLFFIFPCVFAILMFPPGAIFRWLRARRLERIEQQLPDALQMLAGTA

Cnecator LKSGLSRADALRRMDDRLRVKEVTNFVGAVIQAERMGSGLAATLRFQSEQRRTERFQRAEKQAMEAPVKLIFPLVVFIFPVTFIVLGFPPRALVKKMQRKRLMAIEDQMPDALLMMSSAL

Tterpenica MRAGRPRSEALRDLAARLDMAAITNFISTLIQAEATGMSLGPILRAQAEQRRTERFAHAEKLAMQAPVKLLFPLLFFIFPCVFAILMFPPRGVLRWLRKRRVEHIEQQLPDALQMLAGTA

Acaldus LRSGMPRVEVFRRFGDRVAMQELRSLVTAIIQAEERGGSLAPVLKAQAEQRREERFLRAEKAALEAPVKLLAPLIMFIFPIVFIVLTFVPRYVLLYLKKRRFLRLHDQLPDTLLMMASAL

Veiseniae VRAGKARTESLRDMAERLNEPGVTQFVMAVIQAERMGMNLGPVLRAQADQQRSERFLRAEKLAMEAPVKMLLPLIAFIFPCTFIVLLFPPRLLYRQMRKRRLRKFEEQLPDALMMLSGGL

Hneapolitanus MRSGLARAEALKRMAERMDMPQISSFTGTLIQADRVGASLGASLRVQADQRREERFLRAEKLALEAPVKMMLPLVMFFFPLIFLFLAYFPKKFYAWLKQRRIDKIQQQLPDGLMMVSGSM

Phospita LKSGLNRTDALRRLDERQRIGEVSNLVRTIAQAERMGSGLAKTLRFQSEQRRAERFQRAEKQAMEAPVKLVFPLLMFIFPVTFIVLGFPPRQVLAWMRTRRIRMLERQLPDMLLMIAGAL

Dacidovorans IRAGKARAEALRGMAERLNEPSVANFTSAVIQAESMGMNLGPVLRAQADQRRSERFMRAEKLAMEAPVKMLFPLIAFIFPCTFIVLLFPPRLLYRHMRKRRLRKFEEQLPDALMMLSGGL

Taminoaromatica VRAGKSRADALRGFADRIGEPAIANLVSTVIQAENMGMSLGPMLRAQGEQRRAERFARAEKAAMEAPVKMLLPLIACIFPCTFIVLGFPPRLAFGLLRRRRLDLIEQQLPDALQVIAGGL

Smaltophilia LRAGLPRAEALRRMSDRADISQVTSFTSALIQADRVGASLSDTLRSQAMQRREERFLRAEKLALEAPVKMMLPLVLFFFPLIFVVLAYFPRKIYLWLKQRRLDQIQQQLPDGLLMLAGSL

Pphymatum LKSGLKRGDALRRFDERQQIGEVSNLVRTIAQAERMGSGLAKTLRFQSEQRRAERFQRAEKQAMEAPVKLLFPLLVFIFPVTFIVLGFPPRRVLSWMRTRRIETLERQLPDMLLMIAGGL

Ahongdengensis IRAGMGRFDALRLMAERVEVDAVQTFVNAVVQAERTGASVGDVLKGQADQRRTERFQLAEKLAMQAPVKLILPLVAFIFPTTFLMIGFPPRMVYSVLRARRERSFSYTLPDSMAAVASML

Rspathiphylli IRSGLPRAEALKRMSERMEMSQISSFTGTLIQADRVGASLGAALRAQAGQRREERFLRAEKLALEAPVKMMLPLVLFFFPLIFLVLGYFPKKIYRWLRQRRINQIQQQLPDGLMMVAGSM

Psp.T7-7 MRAGVPRIQALQELADRCGLPAVQSLVTALAQADQLGMSLGPLLRSQSEQRRAERFLRAEKLALEAPVKMLFPMVFCIFPCTFLIIGFPPQYVIGRIRKLRLQRFDEQLPDLLQALAGAL

Vparadoxus MRTGVSRMDALNAMAERLDNKHIKSLVSNLTQAESLGASLADTLSAISDQRRTERFQHAEKLAMEAPVKMIGPLVIFIFPVTFIIIFFPPARWFAWQRKKRRQQIESELPDALLFIASAL

Amarplatensis MRAGVSRAAAIKALADRCNSPLARNWAAALAQAESLGISLGPVLRAQAAQCRSDRHVRAEQLAMQAPVKMLLPLIGCIFPCTFIVLAFPPRWTVDRLRRRRADRFEQQLPMALLMLASAL

Aarsenitoxydans MRAGVSRIAAIKALADRSNSQLARNWAAALAQAEALGASLGPVLRAQAAQCRSDRHMRAEQLAMQAPVKMLLPLIGCIFPCTFIVLAFPPRLLVDGLRRRRARRFEEQLPMALLMLASAL

Axylosoxidans VRAGVPRVAAIQAMADRCGSPLVHRWATALAHADALGISLGPLLRAQAAHCRSERHMRAERLAMQAPVKMLLPLIGCIFPCTFLVLAFPPAMLAGLLRRRRVRRFEQQLPMALLMLASAL

Dvulgaris LRAGKSRAEALKNLAARVALEDVNSLVTLIVQADAFGTSISSTLRVYSDTMRTTRFQRAEEIAAKMPVKLLFPLVFCILPALFVTIMGPPFLWVQRKRNARMGRFQRQLPDALDLIARAL

Bexovorus IKIGSSKQEALKEMAARIDIPEITSFVAVLIDAEASGASISQVLKDQSVQMRLERLLRAEKAGARASQLILLPLMMFILPAVFIMVFGPPPIIFKALYERRCSVFVDQMVDALTIMGNGI

Delongatus IQSGLPRKTVLTHLAERNGVESLTTVVGVLLQSIRFGTNIADSLRVHIQSMRTQRRQLAEEAGAKMSTRLTFPMVLLILPALFIVILGPPFLVLLHRRRKKNETMIRQLPEALDMIVRAL

Hmarinus IRIGASRAEALRNLAWRIDLIQISSFCATLIAADSVGASIGPILKALSMEIRQKKSSEVEKAGATAATKILFPMLFLIVPAVFIVVAAPPKPLINYLVDRRINAYSAQMVDALTLLSNGI

Bstolpii IKIGASRAEALRNLAWRIDLIQISSFAATLIAADSVGASIGPILKALSVEIRQKKSSEVEKAGATAATKILFPMLFLIVPSVLMVVFAPPRWIIDYLVEKRIKAYSLQMVDALQLLSNGI

Dphenolica MRAGKMRQDALRNLARRTGIDEMASLVTLLIQTDKFGTSVGSALKVFSDSFRTKRFQKAEEIAAKLPVNILFPLILFIFPSLFVVILGPPCLYLKYLKDKRIQKFKQQLHEALDLIARAL

Dcetonica IQSGLPRKAVLDNLARRNGVESLTSVVGVLVQSIRFGTNIAEALRVHIGSMRTRRRQLAEEQGAKMSTRLTFPMVLLILPALFIVIMGPPAAWLRIRIRSRQKEIFRELPDALDLVLICL

Dmultivorans MRAGKSRQKALRNLGMRVGLSAMDNLVTMLIQTDKFGTSSAQALRVYSDTFRTKRFAVAEEKAAKLGVKILFPLIFFIMPATFVILVGPPFMYLKVKKNARLKKFRQQLPEGLDLIARAL

Cvibrioides LSYLPDRRLAYENLARRTNHPGIKSVATAMIQAERYGTPLGTALRVMAKENRELRLSAAEKKAAALPAQLTVPMILFFLPVLFIMILGPPRWVLGMLAKSRIQKFTEAFADAVDIIVRGV

Atumefaciens LSFLQDRRVALENFGTRTQLETVKSVVQALIQAERYGTPLAQALRVLAQEGRDERMNEAEKKAAALPPKLTVPMIVFFLPVLIAVILGPPRWVLAFILKRRQKKFLEEFPNALDVMCRSI

Aexcentricus LSYLPERRMAYEGLARRTNYPGIKSVCTAMIQAEKYGTPLGTALRVMAKENRELRLSAAEKKAAALPAQLTVPMIVFFLPVLFVVILGPPRWWLSFLAKRRTTKFTANFADAIDILVRGI

Bdiminuta LSYLPDRRMAYEGMAKRTQHPGIKAVATAMTQAETYGTPLGSALRVMAKENRELRLSAAEKKAAALPAKLTVPMILFFLPVLFVVILGPPRWIIGMKAKARMKKFSLAFADAIDILVRGI

Mmaris LSYLQDRRVAYENLAKRTNHPGVKAVCTSLIQAERYGTPLGQALRVMAKENRDMRLAAAEKKAASLPAKLTVPMIIFFLPVLFVVILGPPRWWLGFLRGRRQKQFSGEFANALDVITRGV

Plavamentivorans LSYLQERRMAYENLARRTGLPGVKAVATTLIQAERYGTPLGQALRVMAQENRDMRMAEAEKKAAGLPPKLTVPMILFFLPVLFGVILGPPRWVLSYLIKRRQKAFTEEFANAIDVIVRGV

Nhamburgensis LSYLQDRKVAYENLAKRTGVEGVKSVCLALQQSERYGTPLAQSLRVMAQENRDMRMTEAEKKAAALPPKLTVPMIVFFLPVLFVVILGPPRWLLGFLKTRREKAFLRALPDAVDVIVRGI

Amanganoxydans LSYLPDRKVAYDNLATRTGLDGVRTVCTALVQAERYGTPLGTALRTLSQENRDQRMNLAEKKAAALPPKLTVPMIVFFLPVLFAVIIGPPRFLLSRARKKRFHKFGEEFPNAIDLIVRGV

BbacteriumSG-6C LSYLQDRKMAYENLAKRTGTEGVKSVCLALQQSERYGTPLGQTLRVMAQENRDMRMNEAEKKAAALPPKLTVPMIVFFLPVLFVVILGPPRWLLGFLKKRREAKFLDALPDAVDVIVRGI

Aclevelandensis LSYLQDRKMAYENLAKRTGTEGVKSVCLALQQSERYGTPLGQTLRVMAQENRDMRMNEAEKKAAALPPKLTVPMIVFFLPVLFVVILGPPRWLLGFLKKRREAKFLDALPDAVDVIVRGI

Lsediminis MRVGTARTDALINLCERVNSEYLNSMVGAIIQSERRGTPLANVLEIQVDTIRNKRTAKIEKAASQAAVKILFPLL-FIFGAVMIVIMGAPRVYLKRAWASRLAAIDEQVEEAMVYMANSF

Llitoralis MRVGTARTDALINLCERVNSEYLNSMVGAIIQSERRGTPLANVLEIQVDTIRNKRTAKIEKAASQAAVKILFPLL-FIFGAVMIVIMGAPRVFLKRAWANRLTAIDEQVEEAMVYMANSF

Pcaeni MRVGTPKADALLNLCERVDSDYLNSMVGAIIQSEKRGSPLARVLEIQVDTIRNKRTQKIEKQASQAAVKILMPLM-FIFGAVVVVVMGAPRFWLKMKWNERVKKINEQVEEAMVYMANAF

Bsediminis MRVGTSKSDALLNLCERVDSDYLNSMVGAIIQSEKRGTPLATVLEIQIDTIRNKRTQKIEKAASEAAVKVLFPLM-FIFAAVVVVVMGAPRLWLKREWSARIRQIDEQIEEAMIYMANSF

: : * : : * . * * : *. . : *:: :. . : * : . :

Mxanthus KAGLTFPQAIEHVAREA---MPPLSQEFGLFVKEVKLGVPLEEALINMGRR-VGSDDLELVVVSTNIARQLGGNMAEMFETISTVIRERFRLEGKIDALTSQGKLQGWIVAAMPGVLGMV

Bbacteriovorus KAGSNPQESMKRVVEIM---GNPMSQEFAQVLYQMQVGDSFESALNDLGNR-IPRPDVQMFVTSINILKETGGNLAETFQTIVLVVRERQKVEKKIQALTAQGMMQGIIVTLIPFILMAV

Mmacrosporus KAGLTFPQAIEHVAREA---LPPLSQEFGLFVKEVKLGVPLEEALINMGRR-VGSDDLELVVVSTNIARQLGGNMAEMFETISTVIRERFRLEGKIDALTSQGKLQGWIVAAMPAVLGMV

Mstipitatus KAGLTFPQAIEHVAREA---MPPLSQEFGLFVKEVKLGVPLEEALINMGRR-VGSDDLELVVVSTNIARQLGGNMAEMFETISTVIRERFRLEGKIDALTSQGKLQGWIVAAMPAVLGMV

Saurantiaca KAGLTFPQAIEHVAREA---LPPLSQEFGLFVKEVKLGVPLEEALINMGKR-VGSDDLELVVVSTNIARQLGGNMAEMFETISMVIRERFRLEGKIDALTSQGKLQGWIVASMPAVLGMV

Cfuscus KAGLTFPQAIEHVAREA---QPPLAQEFGLFVKEIKLGVPLEEALINMGRR-VGSDDLELVVVATNIARQLGGNMAEMFETISSVIRERFRLEGKIDALTSQGKLQGWVVAAMPGVLGLV

Dretbaense KAGHAFTTGMKLAAEEF---GDPLGPEFDVALDEINFGVPVPDALRKMTYR-VDCPDLNFFVVAVIMQRETGGNLAEIIESIATLIRERFKFFGKVKALAAEGVLSMWVLIGLPFAIVGI

Paespoeensis KAGHTFGGGMRMVANEF---ADPIGGEFGKTLDEINYGMDVDRALANLQLR-VDVEDLKFFVVSVNIQRETGGNLAEIIANIARLVRERFALFGKVRVLSAEGRISALLLSALPFFIAGV

Ppiezophilus KAGHTFGGAMRMVADEF---EDPIGSEFRTTIDEINFGMDVDRAMANLQNR-VDVTDLKFFIVSINIQRETGGNLAEIISNIASLVRERFVLFGKIKVLSAEGRISAILLASLPFFISGA

Dtoluolica RAGHSFTSSMKLAADEF---GDPLGIEFEETLDEINFGVSVPVALRNLSQR-VDCKEINYFVVAVIIQRDTGGNLAELIESLAHIVRETFKFEGKVRILSAEGRLSGVVLCLIPFLIGLW

OsymbiontRs1 ASGSSLNMALQSVMLEQ---QAPISQEFMLFVREQRIGVDFDASLRNMERR-IPLLDFSMFTAAMRISREVGGDLGEVLSTLADTLRRKSSMEGKIDSLTAQGRMQGIVMTGLPVLLGGL

Dsulfexigens RSGSSLPIALDSLIKES---SPPLSQEFGLYVRERKLGLDRDKAFDNLERR-VPLEDLSLSLSAIRISSEIGGDLAETLESLAETLRKKLVMEGKIDSLTSQGKLQGIVMSTLPLFLIVA

Samazonensis QAGANVTNAIEFMADEM---DAPIKQEFQLFLREQRLGVEFNTALDNVYKR-IPESEFQLVVAGMQISRDVGGNLAEVLVRLSETLRKKIEMEGKITSLTSQGKMQGIVMTLLPVAIGFT

Swoodyi QSGANVSTAIEFMSEEM---QAPIKQEFQLFQREQRLGVEFNTALDNMYKR-IPEDEFQLVTAGMQISREVGGNLAEVLFRLSSTLRRKIEMEGKIDALTSQGKMQGIVMTMLPLFIGFV

Bgladioli RAGASFAVAMESVVSEA---PAPISGELELLMRELRLGIDLDIAMQNIERR-IPVPDFLMVTAAVTISREVGGNLAEALESVARTLGAKLQMEGKIRALTSQGRMQGIVMTLLPLGLMVV

Bsp.CCGE1001 RAGASFPTALEAVVHET---PPPISQEFDLLMREIRLGIDLDIAMRNVEKR-IPVPDFLMMTAAVTIAREVGGNLAEALESVARTLREKLTMEGKIRALTSQGRMQGIVMTCLPLFLMLV

Sloihica QSGANVSNAIEFMAEEM---EAPIKQEFQLFLREQRLGVEFNTALDNMFKR-IPEEEFQLVTAGMQISREVGGNLAEVLQRLSATLRKKIEMEGKIDALTSQGKMQGIVMTLLPVFIGVV

Aferrivorans RGGANLQQAMEGLSRDL---SPPISQELALVVREQRLGVAFEAAIDHLAQR-VPSQDVQLLSSALRISREVGGNLADTVARLGDTIRKRLMMEQKIKALTSQGRLQGIVMTVLPALIIFA

Pxenovorans RAGASFPAALEAVVYET---APPISQEFDLLMREIRLGIDLDIAMRNVEKR-IPIPDLLMMTAAVTIAREVGGNLAEALESVARTLREKLQMEGKIRALTSQGRMQGIVMTCLPLFLMLV

Csp.GA3-3 RAGASFPMALESVVAES---RPPISQEFDLLMREVRLGVDLMDALRNMEKR-IPVPDFLMVTAAITISREVGGNLAETLESVARTLREKHQMEGKIRALTAQGKMQGLVMTGLPLFLIVV

Tsp.28 RAGLSLPAAIRQVGSEL---APPLQQELLLLQHEQRLGVSLDDALENLALR-VPAQPVKLMVSAMRIASETGGGLAETLERTATTLRSQHAMELKIRALTAQGKLQAWVVGLLPLFLLWV

Cnecator RAGASFPMALESVVAES---RPPISQEFDLLMREVRLGVDLMDALRNMEKR-IPVPDFLMVTAAITISREVGGNLAETLESVARTLREKHQMEGKIRALTAQGKMQGLVMTGLPLFLIVV

Tterpenica RAGLSLPAAIRQVSAEL---AAPLQQELLLVQHEQRLGVSLDDALENLARR-VPAQPVKLMVSAMRIANETGGGLAETLERTASTLRSQQAMELKILALTAQGKLQAWVVGLLPVFLLWV

Acaldus RGGANLGQTLEGLAKDM---SPPMNQELSLVVREQRLGVPFEEAVSHLAER-VRSQDFDLVVSALRINREVGGNLADTLQRLGETVRRRLMMEQKIRALTSQGKLQGIVMTALPVLIILA

Veiseniae RAGLGLSSAMAQLVAES---GPPLSQEFSLMLREQRLGVTLEQALSNLNHR-MPTQTTVLITSAMRIANETGGGLAETLERTSATIRSRLRMQAKIGALTAQGKLQAWVVGALPIALMLI

Hneapolitanus RAGLGFTAALESLARDT---DPPLAQEFALVLREQRMGVKLEDALLHFNDR-VPVQDVTLFVSAVNISREVGGNLAESLTSLASTLRRRLIMEGKVKSLTAQGRLQGIVMALMPVGLLTF

Phospita RAGASLPIALESGVQES---APPISQEFDLLLREIRLGIDLDIAMRNIEKR-IPVPDFMMVTAAVTISREVGGNLAEALESVARTLRDKLQMEGKIRALTSQGRMQGVVMTCLPLFLMMV

Dacidovorans RAGIGLSSAIAQLVAES---RPPLSQEFSLMLREQRLGVSLEQALNHLSLR-VPTQSTVLITSAMRIASETGGGLAETMERTASTVRSRLQMEAKIGSLTAQGKLQAWVVGALPLVLMLI

Taminoaromatica RAGVSMTVALQQLVREG---RPPIAQEFDLTLREHRLGIPLDEALDHLAAR-VRMPSLTLVIAAMRIANETGGSLAEALERAALTVRSQLAMEGKIGALTAQGKLQAVVVGMLPLALLLV

Smaltophilia KAGVGFNPALEALAHDG---LPPLAQELALVLREQHMGVRTEEALDNFAAR-VPIADVKLFVAAVSISREVGGNLAESLSTLAETLRRKLIMEGKVKALTAQGRLQGIVMAMLPAGLVGF

Pphymatum RAGASFPIALESAVQES---VPPISQEFDLLLREIRLGIDLDVAMRNIEQR-IPVPDFMMVTAAVMISREVGGNLAQALESVASTLRDKLQMEGKIRALTSQGRMQGIVMTCLPLFLMMV

Ahongdengensis RAGSNLQSALELMVKES---SGPIRQEFGLLLRELKMGVDFNEALDNMLER-MPGEDLRMLTAGMKIAREVGGSLADVLARLAETLRRKLEIEGKIKALTAMGKAQGYVMALLPLAVGFA

Rspathiphylli RAGLGFTPALESLARDV---EPPLAQEFALLLREQRMGVKLEEALEHFNDR-VPIQDVTLFVSAVGISREVGGNLAESMASLADTLRRRLIMEGKVNSLTAQGRLQGIVMAMMPVGLIGF

Psp.T7-7 RAGAGLQSALKHIVAQS---PAPLSQEFGLMLRQQRMGIGFEQALADLYRR-MPFEGTGLVVSALTIAAQSGGNLAETLESIAATLRARLRLLSRVQALTSQGRLQAWIMAGLPPVLAIV

Vparadoxus RAGSALSVAIQVLVRDQ---VGPLGQEFGLVLKEQRLGVSFDDAIQKMSQR-LGIPDFVLVVVALRVSKEVGGNLSEPLQVLAETLRRKAILEGRIKALTAQGRIQGLVMTLFPLLLMGV

Amarplatensis RAGVALATGLRHVVEQS---GAPLAQEFGLMLREQRMGVPWDAALENLHAR-MPADSTSLVVAAMRIAAQTGGNLAEALESIAQTLRAQLQLQAKLRALTSQGRLQAWIVGALPLLLLAV

Aarsenitoxydans RAGVALATGLRHVVDQS---GAPLAQEFGLMLREQRLGVPWDAALDNLQAR-MPADSTTLVVAAMRIAAKTGGNLAEALESIAQTLRARLQLQGKLRALTSQGRLQAWIVGALPLLLLAV

Axylosoxidans RAGVALMPALRQVVAQG---EAPLAQEFGLMLREQRLGVPWDEALARLNTR-MTAKSTTLMVAAMRTAAQTGGNLAEALDSIAQTLSARLQLQARLRTLTAQGRMQAWIVGALPVLLLIA

Dvulgaris KAGHAFNQGMRMVADEF---ADPIGPEFQKTLDEINFGIPADAALYNLTRR-VDCPDLKFFVVSVNIQRETGGNLAEIVTNIARLIRERFKLAGRVRVLSAEGRLTAWILFVLPFGIGFI

Bexovorus KAGSNPQQSMQRVIEIM---GNPVSAEFSQVITQTQFGQSFEEALNDLAAR-IPKADVQMFVVAVNILKETGGNLSETFTTIVTTIRERQKLEKKISAMTAQGIMQGIIVSCIPFVLMAV

Delongatus RVGQSVDNALLEVSRSC---PPPLGPEVRIVYEEVTLGLPFIGALRNFEARSAGLPDIKLMVTAFVIQHETGGNLSRILDNLADLIRKRDTLRRQVRALTAEGRSSALVLGFLPLVVGLF

Hmarinus RAGLSVPQAIGMVVDEM---PAPVSQEFNMILQQNRIGVPLEECFDNLSKR-IPTEDNDMFVSSINILRETGGNLAEVFDTIVDVIRERVRLKQKIDTFTAQGMFQGLTIFLMPYGIGLI

Bstolpii RAGLSVPQAIGMIVEEM---PAPISQEFNVLLQQNRIGMPLEECFENLAKR-VPLEDNDMFVSAVNILRETGGNLSEVFDTIVDVIRERVRLQQKVDTYTAQGMFQGMTIGAMPYLLGFV

Dphenolica RAGHSFTSSMKLAADEF---GDPLGIEFEETLDEINFGVSVPVALRNLSRR-VDCKEINYFVVAVIIQRDTGGNLAELIESLAHIVRETFKFEGKVRILSAEGRLSGVVLCLIPFLIGLW

Dcetonica EAGLNFDLSLYRVSRELKRVSPVMSEEFGQYFLEIQSGLPRKAVLDNLARR-NGVESLTSVVGVLVQSIRFGTNIAEALRVHIGSMRTRRRQLAEEQGAKMSTRLT------FPMVLLI-

Dmultivorans RAGHAFSNGLKLASEEF---DDPLGTEFKETIEEINYGLSTAEALKNLARR-VDCEDLRFFVIAVILQQESGGNLSELIENITRLIRERFKFYDKVQALTAEGRLSGLIIGITPFFLFSY

Cvibrioides KSGLPVHDCLKIIGKES---PEPLAGEFRILTENIGMGVPMDAALEKMYER-MPTSELRFFSIVLAIQQKTGGNLAEALGNLSTVLRSRKLMKEKIKALSAEAVASAFIIGCLPPGVVTM

Atumefaciens KSGLPLNDAVRLIASDG---QEPVKTEFQRVIDALQVGIGIPQGIERMMLT-VPLFEVSFFSTVINIQAQAGGNLSEALSNLSKVLRERRKMRAKVNALSMEAKASAVIIGALPFIVMLL

Aexcentricus KSGLPVHDGLKIIAKEL---PAPLGPEFQRLNENISIGMGLDMSLEKMCER-MPTPELRFFTIVIAIQQKTGGNLAEALGNLSHVLRSRRMMREKIKALSSEAIASASIIGVLPPGVGIM

Bdiminuta KTGLPVHDCFKIIARES---PEPLAGEFRTLVEGMGVGLTLAQALDKMYER-MPTPELKFFAIVIAIQQKSGGNLAEALGNLTTVLRARRMMVEKIKALSSEAIASAGIIASLPPAVMIL

Mmaris KSGLPLNECLKILSVES---PEPVKTEFAKLVEGIAVGVSLPDGLDRMCER-MPLPELNFFRTVLIIQQKTGGNLAETLGNLSVVLRSRKMMREKIGALSSEAKSSAAIIGSLPPGVMGI

Plavamentivorans KSGLPVNDCLKLIATES---PEPVRTEFQGIVEGQRVGVTLEQGLAKIYER-MPLPEVNFFQIVLAIQQKTGGNLSEALGNLSKVLRERKKMRGKIQAMSQEAKASAGIIGSLPPGVMLL

Nhamburgensis KAGLPLFESIKVVAADS---PEPLKSEFAAIIETQAIGMPLGEACARLYER-MPVPEANFFGIVIAIQQKSGGNLSEALGNLSKVLRDRKKMAEKIQAMSMEAKASAAIIGSLPPVVMML

Amanganoxydans KSGLPLNDTLRIVSGET---LDPVRSEFRKIIESQQMGVPISEAVEKLYRN-IPTPEANFFAIVIAIQSQAGGNLSEALGNLSNVLRDRKKMRAKVQAMSMEAKASGGIIGALPVVVAGL

BbacteriumSG-6C KAGLPLFESLKVVAADS---PEPLRSEFNAIIETQTIGMPLGEACQRLYER-MPLPEANFFGIVIAIQSKSGGNLSEALGNLSKVLRDRRKMAAKIQAMSMEAKASAGIIGALPPIVMIM

Aclevelandensis KAGLPLFESLKVVAADS---PEPLRSEFNAIIETQTIGMPLGEACQRLYER-MPLPEANFFGIVIAIQSKSGGNLSEALGNLSKVLRDRRKMAAKIQAMSMEAKASAGIIGALPPIVMIM

Lsediminis KANPSMPEAIQDVCNAM---GPPISQEFGVLLKEYRLGTPLDQALVNLQRR-VPSRNLELAISALVIGRTVGGNIPEILSQISGTIRESFRLERVIDTQTAQGKMQAWVMGLMPAVVIGV

Llitoralis KANPSLPEAIQDVCNAM---GPPISQEFGVLLKEYRLGTPLDQALVNMQRR-VPSRNLELAISALVIGRTVGGNIPEILSQISGTIRESFRLERVIDTQTAQGKMQAWVMGLMPAVVIAV

Pcaeni KANPSLPEAIADVTNSM---PPPISQELQVLLREYKLGTPLDQCLIRLQQR-MPARNLELAISALLVGRTVGGNIPQILDDISSTIRESYRLERVIDTQTAQGKMQAWVMGAMPAVVIGV

Bsediminis KANPSLPEAMADVTTAM---PAPISQELQVMLREYKLGTPLDDCLIRLQQR-MPARNLELAISALLVGRTVGGDIPKILEDIGSTIRESYRLERVIDTQTAQGKMQAWVMGSMPAIVVTV

. . : *. * . * .: . : * :

Mxanthus LNYMRPDLMEPMMDHIFGWILVIIIAIMEVMGILIIRRIVNIDI----------------M-------------------LKGKTPLVVAVAAQDVPEGGDPLLWSQFETTKAAE-----

Bbacteriovorus FLVIDPAFIKPMFNTTLGLVLLAAMLGLQIIGGVLIKKLVTIKV----------------------------------MGSNETRNLWLSVAKEDIAEMGQMVVKNNLLTPGPDT-----

Mmacrosporus LNYMRPDLMEPMMNHIFGWILVIIIAIMEVMGILIIRRIVNIDI----------------M-------------------LKGKTPLVVAVAAQDVPEGGDPLLWSQFETTKAAE-----

Mstipitatus LNYMRPDLMEPMMNHFFGWILVVLIAIMEVMGILIIRRIVNIDI----------------M-------------------LKGKTPLVVAVAAQDIPEGGDPLLWSQFETTKAAE-----

Saurantiaca LNSMRPDLMEPMMDHMFGYVLVTVIAIMEILGILIIRRIVNIDI----------------M-------------------LKGKTPLVVAVAAQDIPEGGDPLLWSQFETTKAAE-----

Cfuscus LNYMRPDLMEPMMDHWFGYVLVTIIALMEVMGVLIIRRIVNIDI----------------M-------------------LKGKTPLIIAVATQDLPEGGDPLLWSQFETTKAAE-----

Dretbaense LTFMNPEYITLLFEETMGHIMIAGALIMMIIGYFFMRNIVKIDV---------------------------------MGRLRALIPIALAVAALDISAGNEPIVDHRLAPTDIKSG----

Paespoeensis LYFINPDYISLLWTNELGRSMAWGAVASMIVGIVVMRRMVKIQV--------------------------------MSRSARALIQIGISVALTDLRPGNEAVTEAKLADASVIGG----

Ppiezophilus LYMLNPEYMSRLWTTELGRTMTWGAIIAMSVGIVVMRKMVKIKV--------------------------------MSKSTRALLQIGIAVAREDIHRGNEAVTKSKLADPSIMGG----

Dtoluolica LQISSPGFMKPLFTEPIGRILLGVAGVMMIVGMFVIKKIVKIEV---------------------------------MGNIRTFVPILLSVAKAGIPWGGEPVLEYRLAPVSMKTG----

OsymbiontRs1 LFALEPEAMSKLFTTPMGWGLLSVIVVMEVLGYIFISKITSIDV-------------MATSSK------------------KTITLLVVAVASKDLLKGGKMLLASYVGADFPL------

Dsulfexigens LMKLEPAAMGQMFTTKIGWMVLATIVCMQVLGFIAIRKITAIDV-------------MENTEQ-------------NKIKNKTWKLFTAAVASRNLPTGGKMLAQDYIDLKIPK------

Samazonensis LYHMEPVSMGRILTEPVGWAVIAVSSLLLFGGYMTIKKIITIDV-----------MQSKRIDF-------------------NWVLLGIAVATQELRKGGRPLISAYLPGFK-TR-----

Swoodyi LYHMEPKSMGRILTEPMGWALMALIFIMLTSGYLSIRKIVAIDV-----------MQIKSFDF-------------------NWVLLIIAVATQELRKGGRPLMGTYLPGHK-AE-----

Bgladioli LNFMEPEAMAPLFHERVGWLTLAVIAVMEFLGYKAISKITNIDV-----------MIFNRINY-------------RSLLANTWLLIGIAVPRADVPAGGRPLRLQDVDATRAR------

Bsp.CCGE1001 LRFMEPKAMAPLFTQPVGWATLAVIGVMELLGYFSISKITNIDV------------MLKKIKI-------------RSLLSNSWVLLFAAVPARDLPAGGLPLRAGDIDALRGR------

Sloihica LYHMEPSAMGRILTEPMGWALIALVIIMLTSGYLSIRKIVAIDV-----------MKLKSIDF-------------------NWVLLVIAVATQPLKKGGRPLIETYLPGMR-VA-----

Aferrivorans LLQIEPQAMGALFNTTRGWAVLAIAILFEVFGYLWIQKIVSIEV------------MSTPIKP---ESTPPVLQKK-SRALTGWILLVVAVPIENLPAKGKPLTLSMFSSTE--------

Pxenovorans LRFMEAKAMAPLFSEPVGWATLAVIGVMECLGYFWISRITNIDV------------MLKKIKF-------------RSLLANSWVLLFLAVPAQDVPVGGLPLRAGDIDALRGR------

Csp.GA3-3 LNQMEPVAMAPLFNSPIGWGTLSVIAVMELLGYKAISKITHIDV------------MLKKIKI-------------HSIIGNPWVVLVIAVPVRNAAPGGQPVRRADVEALQSR------

Tsp.28 LTHMEPEAMALLWTTRMGWAVLAVVLVMELTGVLLIRRIVSIDI-----MTPTNTP--PRA-------------------RKHWVVLAFAVAKSNLSGGGEPILWAQLEGNRAP------

Cnecator LNHMEPVAMAPLFSSPIGWGTLSVIAVMELLGYKAISKITHIDV------------MLKKIKI-------------HSIIGNPWVVLVIAVPVRNAAPGGQPVRRADVEALQSR------

Tterpenica LNRMEPAAMSQLWTTPLGWGVLAAVLVMEFIGVLLIRRIVAIDI------MPPNTPITRNT-------------------RKNWIVLAVAVAKTSLPAGGEAILWAQLEGRRAP------

Acaldus LLRIEPAATGALFDTYRGWAVLAIAIVLEILGYWGIRKIVSIEV------------MATPLPP---EFEPGTVQKKPARAIGGWLILAVAVPVQDIAPGGRPILASYLSGRR--------

Veiseniae LNEMEPQAMSMLWHTRVGWATLAVIALFEFLGVYVIRKIIAIDV----MKLPLSRLATLRP-------------------GKTWLALGAAVAKRELKKGGEMLLWSLMQTRKAA------

Hneapolitanus LTLAYPDTMHDLYHTPLGWGIIAVAVVMEYLGYRMSLKIVTIDI----------------MP----------------KVNRNFLYVAVAVPVHNLKVGGAPISAMAMEPIANH------

Phospita LRAMEPVAMAPLFTTPIGWETLAVIAVMEFLGYQSIARIVRINV------------MFRKIKF-------------HSLLGNAWFLLLVAVPRRDVAAGGRPLRMSDIDALRGR------

Dacidovorans LDRMEPEAMSMLWHSRVGWGALAVIACFEAMGIYVIRKIVAIDV----MNSALARITALRP-------------------NKTWVALAMAVAKRNLSKGGDILLWNLLEGDKVP------

Taminoaromatica LDRMEPEAMGFLWHSPPGWATLAVIATLEVLGVLLIRRIVAIDV------------MLKRV-------------------PRNLIILAVAVAKTDLAVGGEPLVWSQLEERRPA------

Smaltophilia LVLFYPETMNPMFHTFIGWCVMALIGVLEYLGYRMCRKIMTIDI-------------MKALPL---------------KANRNVVFIIIAVPKTDMEPGGAPLGASALVPLYDQ------

Pphymatum LRAMEPVAMAPLFTSPIGWATLAVVAAMELLGYASIARIVRINV------------MFRKIKF-------------HSLLGNAWLLLLAAVPRRDVPAGGRPLRTSDFDAPRGR------

Ahongdengensis IAQIEPEAMDKLFTTMAGWAVCAVVVVLELLGFWFIKKIVSIDV----------MKKYTAI-----------------------IMLVVAVASRDLYPGGTALISAFIAGMQGVG-----

Rspathiphylli LSFAYPDTMHEMYHSYVGWAVIAVAAVMEYLGYRMCRKITSIDI------------MERRMP----------------AVNRNFLYLGGAVPVHDLEKGGAPIGGSAVDRVTDH------

Psp.T7-7 LHYLDPDSMRALWETPAGWSVVAAIVTMEVLGIWFIRRIVAITI--------------MRLP----------------VFNRSLAMLLLAVAAYDLPAGGDAVLEVHTTGQDHA------

Vparadoxus LYLMQP-TMSLLFTTTIGWVVLTVIAIMLMLGYAMIRKIVAIDIMKSSPGLLKFKQSVKRLSP-----------------------LIGAVANRALGDGGKPLLASFFSAPRKV------

Amarplatensis LDRLEPEIMGLLWHTPMGWGVLAIVAVLETAGIVLIRRIIRIDI--MGIVKALLIHGMRRL--------------------RRVGVYGLAVAAYDLPAGGEPLLKTHLTF--TAPA----

Aarsenitoxydans LDRMAPETMGLLWHTPMGWGVLAVVGVLEVAGGLLIRRIMRIDI-----MKGGILRGAARL--------------------RRASVYVLAVAAYDLPAGGEPFLRGFLSF--NAPV----

Axylosoxidans LDRLEPDIMMPLWHTPAGWTVLAVLGGLETAGVLLIRRIVRIDI-----MMRLLGWGLRAL--------------------RKMAVPLLAVAARDLPVGGDPVLSGCLARADSASP----

Dvulgaris INLLNPDFMSALYATPEGNMLLTASLFQMAIGGIALKRLTTIRV----------------------------------MRASSLIQISLAVAAADVPRGGEPITEARLLQDGASHG----

Bexovorus FYFLDPTHLEPMFGTTLGLLLLVGMLGLQIIGGVMIKKIVTIKV----------------------------------MGPNESRNLWISVAREDIRHMGQTLTKNQILELGPET-----

Delongatus FWLVRPVYIQVLFVHPVGQKMLLLAILLEFFGFLVMRLLIRITP---------------------------------MRKLRALIAMFVATSSPETKEGHQDQLPVNQQPSAFSSEDGIH

Hmarinus YFVSDPESVKPMFTHPLGIILTLAALAFDFAGGFVILKIVKIKV-------------------------------------MNTRALTLAIAKVDIKELGEQITKPRVSWPDERS-----

Bstolpii YFLQDPKSMTPLFTTPLGLVFLFVALIFDIAGIYVIMKIVKIKI-------------------------------------MNTRALTLAVAKNDIQELGEQITKPRVTYPGIKT-----

Dphenolica LQISSPGFMKPLFIEPIGRILLGVAGVMMIIGMFVIKKIVKIEV---------------------------------MGNIRTFVPILLSVAKAGIPWGGEPVLEHRLAPTSMKTG----

Dcetonica --------LPALFIVIMGPALINIYERIQGGGF---------------------------------------------------------TPAPDLPEGGQDKLPA----TAFAPTMGIG

Dmultivorans FYYMNPDYLELILTRDIGRLMLIVSGVMLLIGLWIIKKIVDIEV---------------------------------MGRTKALIPMILAVAAANIPWGNIPILESMLASADVKTG----

Cvibrioides ISVTSPEYMKPLFTDPRGHLMLLAGAVLMTCGILLMRKMINFKF----------------------------------MNPVRIIIVLVAVAKRDLAIGGEPVVARKIVRGG-EGG----

Atumefaciens VHFTSPDYLSVLFTDMRGHIILGVSALWMLIGIFIMRQMINFDI----------------------------------MKPSRIVILSVAVSSVNLPVGGEPVRPEKVVDSS--TR----

Aexcentricus IYITNPGYMGPLLTDPRGNFMLLGGAVWMLCGIFMMRKMINFKF----------------------------------MKASRFVVVGIAVAARHLKVGNEPIVARKVVRAD-AGG----

Bdiminuta VMLSNPSYMMLMFTDIRGQVMLMGAALWMATGVFVMKRMISFKF----------------------------------MKPAKIAVICIAVAARDLEPGGEPLVARKIVRAG-DSG----

Mmaris VYATTPSYMGQMFTHPTGQLMLLGGLTWMFIGIMVMRGMINFKI----------------------------------MNAVRIAILAAAAARRDVEIGGEPITGRRLVQPG-DAG----

Plavamentivorans IYFSSPGYMDVLFSTTAGNMIIVGGIMWMAIGVLVMKKMIDFKF----------------------------------MNPVRIGVLVLAVASSNLQLGGEPVTSEKLVTLT-GAG----

Nhamburgensis VYLTTPDYIAMLWTHPTGRLMLAGCVLWMSAGIFVMKKMINFDF----------------------------------MNTARIVVLTIAVAKSDIPLGGEPIRELKLVSAK-GSG----

Amanganoxydans VYITTPDYISVLFTTGTGHLILAVSGLWMATGVFVMKKMISFDF-------------MSAITVVNSRMVAASFARSSPMNLARMAVVAVAVTSRDLSMGGEPVRESKLIRSD--QG----

BbacteriumSG-6C VYIMVPEYISLLWTHPTGRLMLAGCAMWMGMGILVMKKMINFDF----------------------------------MNTARIVVLTIAVARSDIALGGEPIRETKLVKAN-GSG----

Aclevelandensis VYIMVPEYISLLWTHPTGRLMLAGCAMWMGMGILVMKKMINFDF----------------------------MWKVKRMNTARIVVLTIAVARSDIALGGEPIRETKLVKAN-GSG----

Lsediminis FYKMDPTLIQPLFETFVGYIVLGLAGVCNIIGVVMILKIVQIDV----------------M-------------------AKKKL-LIAAVAARDIASGGSMILTSDFAVAEVAR-----

Llitoralis FYKMDPTLIQPLFETFVGYIILAAAGVCNIIGVVMILKIVQIDV----------------M-------------------AKKKL-LIAAVAARDIQAGGSMILTSDFAVAEVAR-----

Pcaeni FYLMDPELIEPLFSSWIGYIVIAIAVVLNIIGVALILKIVNIRV----------------M-------------------AKKKL-LIAAKAARDIPAGGAMILTSDFNVKEVSS-----

Bsediminis FYLMDPELIGPLFNTLTGYLILSVAIVLNIIGVILILKIVNIRV----------------M-------------------AKKKL-LIAAYSATDVPEGGDMLLSSDIASVQSAN-----

: * * .

Mxanthus -------RLSSKVQKKARALTIEAKHTTSVGGWIRPNDHVDLQNVIVVASLMVLPEEAEILVLAVE----LGQLT-LSLRNE--D-----D

Bbacteriovorus -------GISLQVAPSKRAVTIPVDEVRGVAKLIRPGDRIDMNDVVVLATVEATPKEAQDLFYILST--APGNLF-MALRNP--S-----D

Mmacrosporus -------RLSSKVQKKARALTIEAKHTTSVGGWIRPNDHVDLQNVIVVASLMVLPEEAEILVLAVE----LGQLT-LSLRNE--D-----D

Mstipitatus -------RLSSKVQKKMRAMTIDAKNATAVGGWIRPNDHVDLQNIIVVASLMVLPEEAEILVLATE----LGSLT-LSLRNE--D-----D

Saurantiaca -------RLSTKVQKKGRAVTIEAKSTTSVGGWIRPNDHVDLQNVIVLASLLVIPEEAEILVLASE----LGNLT-LSLRNE--D-----D

Cfuscus -------RLSTKVQKKVRAVTIDSRVTTSVGGWVRPNDHVDLQNVIVLATLMVIPEEAEILTLASE----LGNLT-LSLRNE--E-----D

Dretbaense -------GVSALISKGNRAVSLGGNKVLGISGFVLPGSRVDLDNVKILATLEVTPEEAEILTHIRN----QGSLQ-MALRNP--T-----D

Paespoeensis -------GVSALIEPGKRAMSVKGNAVMGLAGFVRPGDRVDLERVKVLATLELTPDESERLALAAT----QGTLN-FALRNA--T-----D

Ppiezophilus -------GVSALIEPGKRAMSVKGNSVMGLAGFVRPGDRVDLERIKIIATLELSPAESERLALAAT----QGTLN-FALRNE--Q-----D

Dtoluolica -------GVSAILESGKRAVSVKGNNVLGIAGFINPGNRVDLENILVLATLEVTPDQGERLTLAAT----KGRLQ-FALRGA--T-----D

OsymbiontRs1 -------DFSDTLTIKRRAMTIQVDEMSTFTGLLRPGNRIDLENVEVLTTLNVTAKEAAILASAQD----KGDLL-VLLRNR--K-----D

Dsulfexigens -------DFSGTIQSGHRAITIQVDEVNSISGMIKPGNSIDLEDVLVLATLEVTPKEAAIVAIAES----RGELI-SILRNS--N-----D

Samazonensis -------QFSDLLQEGQRAVTIDINEENSTAGMLVPSDLVDIEKAQVLATLALSTEDAIRVSLAKG----RGKFV-TLLRNK--A-----E

Swoodyi -------QFSDILKEGQRAVTINIDELNSTAGMLVPSDHIDLENVTVLATLALSVHDAARVTLAKE----KGKFV-TFLRNQ--T-----E

Bgladioli -------DFSDTLAAGMRALTLPIDAINSTDSMLRPGNRVDMPGVRVLATLQVPVAEAARIALAQK----LGGLR-LVLRNS--K-----D

Bsp.CCGE1001 -------DFSDILPAGQRAVTVEIDTVNSTALLVRPGNRVDMPNVLVLATLQVPAEDAGRIALAQK----IGGLR-LILRNA--D-----D

Sloihica -------QFSDILKEGQRAVTIDIDEINSSAGMLVPSDHIDLEDVTVLATLALSVNDAARVSLAKQ----KGDFV-TLLRNQ--K-----E

Aferrivorans -------SPADILDSQHIALTISVNSENSMDKMLRPGDRVDGQNLKVIATLEVTPLQAQKILVAQK----SGEIR-LDLRGN--------D

Pxenovorans -------DFSDVLRPGTRALTVEIDTVNATALLLRPGNHVDMPNVLVLATLEIPVDDVARVALAQK----IGGLR-LILRNS--E-----D

Csp.GA3-3 -------DFSDLLKPGKRAFTIEVDMVNSTAKMLKPGNHVDMSDMLVLATLMVSPEQAARLALAQK----VGSLR-AVLRNA--A-----D

Tsp.28 -------SFSARLQTGQRAVTVPVDEISSLSGMVEPGDRIDLQSVAVLATLDTSPEDAKRVIAARE----IGRVT-ALLRAP--G-----D

Cnecator -------DFSDLLKPGKRAFTIEVDMVNSTAKMLKPGNHVDMSDMLVLATLMVSPEQAARLALAQK----VGSLR-AVLRNA--A-----D

Tterpenica -------TFSARLQSGQRAVTVPVDEISSLSGMVEPGDRIDLQSIPVLATLDTSPEDAKRVIAARE----IGRVT-ALLRAP--G-----D

Acaldus -------TLADIVDPDKLAMTFTVDNISTLDGMLQPGDHVDEHDLKIIATVALSPEQIQKLVMAKR----VGELQ-LVLRSH--------D

Veiseniae -------TFSARIEIGRRAITVHVDEINSISGLLEPGDAIDLQNVQVMATLDTTQAQAQSVIVARE----AGKIT-ALLRNP--Q-----D

Hneapolitanus --------FSDIIKPDRVAYTIDVSETNSISGLIVPGDHIDLSDVLVLATLELTPKNAQRIGIAKK----MGQLL-VMLRSP--D-----T

Phospita -------DFSDMLPAGQRALTLEVDAVNSTASMLRPGNRVDLPDVLVLATMQVPAADAPRVVLAQK----MGTLR-LILMNA--D-----D

Dacidovorans -------TFSARIEVGRRAITVHVDEINSISGLLEPGDAIDLQNVQVMATLDTSKAQARSVIIARE----AGKIT-ALLRNP--K-----D

Taminoaromatica -------TLSAQLAPGRRATTIPVDETSSLSGLLRPGDRIDLQGVRVLATLDLSEEEATRLVAARA----LARLT-AVLRHP--R-----D

Smaltophilia --------FSRVIAKGNVGYTLQVDETNSISGMIAPGDHVDLEDINVLATLELTPAQAQRLTVAAK----AGSMR-VMLRQV--E-----D

Pphymatum -------DFSDMLPAGQRALTFEIDAVNSTASMLRPGNRVDLPGVLVLATMQVPAADAPRIVLAQK----MGTLR-LILRNAADD-----D

Ahongdengensis -------SFSELLRPGERAITVDASPVASSENLLQAGDHIDLDNVTVLATVGVPVEDVAKMLRARA----AGELS-FLLRHP--E-----D

Rspathiphylli --------FSNVIEPGKVAYSIQVDESNSVSGLIVPGDHLDLGDVPVLATLGLAPNDAQRLGMAMK----VGELR-VMLRPA--G-----N

Psp.T7-7 -------AFSSHLSAGRRAISMPVDAINSVSGLLQPGDLIDLQGVLVLATLDASPEDAVKLVAARQ----GGTIT-AVLRPP--Q-----D

Vparadoxus --------FAQEIEQGVRAITIPVDEISSISGMLRAGDRIDLQDVEVRATVAIQPQDAQKLILAQR----LGKII-ATLRNP--E-----D

Amarplatensis ------PALASRLRAGQRALTVAAGDLGGLAEMLRAGDLIDLQGMRVLTTLAADPDEAVRFVAARQ----SGTLT-AMLRHR--D-----D

Aarsenitoxydans ------PTLSSRLRAGQRALTVAAGDLGGLADMLRAGDSIDLQGMRVLTTLAATPDEALRFIAARQ----AGALT-VMLRNR--D-----D

Axylosoxidans ------PALAARLDPGQRALQVPVSDLGTVADLLRVGDLVDLQGVRVLATLAATPNQAMRYLAARR----AGTLT-GLVRNR--A-----D

Dvulgaris -------GVSTLIAPGMRAVAVKGNKVLGLSGFIRPGNRVDLENVRVLATLEISPPQAEPLALAAS----RGELH-FALRNP--A-----D

Bexovorus -------GMAPQVSPGKRAVTIPVSDDRANARLIRPGDRVDMQDVVILATIEARIEEAQDLVYLAAT--NSSSLY-FLLRNP--Y-----D

Delongatus AYLPQSLELETLIAPGMRAITLEVTATDGVGGFFRPGDRVDFENLKIVSTLEVTPKQAEELTVLADSKQDKNMLR-LISRNK--N-----D

Hmarinus -------GLSRQVAVGKRAISLDVSERASVGKLIKPGDRVDLQDVLILSTLELDPYEVQKLVFILSY--NSGTPPYLALRNN--T-----D

Bstolpii -------GLSRQVSVGKRAVAINITEKDAVGRLIKPGDRVDLQDVLVLSTLELDPYDVQKLAFMQAY--GSGTLS-LSLRNN--A-----D

Dphenolica -------GVAAILESGKRAVSVKGNNVLGIAGFINPGNRVDLENILVLATLEVTPDQGERLTLAAT----KGRLQ-FALRGA--T-----D

Dcetonica AFRPQPRDLDTFIAPGMRAFTLEVSPTDGVDGIFRPGDRVDLQDIRIVATLEVTPEDAERLTVLADSKQGRNMIR-LISRNS--R-----D

Dmultivorans -------GMAVVVSPGKRAVAVRGDKVLGLSGLVHPGNRVDLEKIPVLATLEVTPEESEKLALAAT----RGQLH-FALRNI--Q-----D

Cvibrioides -------YLSVVLSPGARAMAVPVTSETAAGGFILPGDRVDLQNIRVLATLEVRPAEAEALTRAKA----SGPVT-LALRAY--T-----D

Atumefaciens -------IMSSLLPAGKRAVATEISVSTGAGGFVLPNDRVDLNNVRVLATLELTPEQAKIITVAQQM---ADRLT-LALRSV--ADAQEND

Aexcentricus -------FMAVMLAPGMRAMAVPVTVESTAGGFILPGDRVDLRNVKVLATLEVRPEEGEALAQAKA----QGPLS-LMLRSY--A-----D

Bdiminuta -------YMAAYLEPGMRAMAIRVTVETAAGGFILPGDRVDMRNVKVLATLELTGRDAEVLALAKS----EGELS-LVLRSY--A-----D

Mmaris -------FMAAVLTPGMRAVAVPISAETGAGGFILPNDRVDVENARVLATLELTPDQARAVSVAVA----RGEIA-LVLRSL--T-----D

Plavamentivorans -------FMSALIEPGMRATAISITPETSAGGFILPNDRVDLRNIRVLATLELTPAQVEVVSVAEA----EGRIG-LSLRSM--AE---QD

Nhamburgensis -------FMAAILPTGMRAISTEISPETGAGGFILPNDRVDLSNVRVLATLELKPEQAEMLARSRQ----MGTLS-LALRSL--A-----D

Amanganoxydans -------FMSAVLPAGKRAVAVQIAAETSAGGFVLPNDHVDLRNLRVLATLEVDPEQAEALTAAQQI---ADRLV-LSLRSL--A-----D

BbacteriumSG-6C -------FMAAILPTGMRAISTEISPETGAGGFILPNDKVDLTNVRVLATLELKPDQAESLARARQ----AGTLS-LALRSL--V-----D

Aclevelandensis -------FMAAILPTGMRAISTEISPETGAGGFILPNDKVDLTNVRVLATLELKPDQAESLARARQ----AGTLS-LALRSL--V-----D

Lsediminis -------TLSGRIPAGERAMTIPVDAISGVSGLLRPGDRIDLQNVTLLATLSLTPSEAELLVISQT----RGKLM-LLLRHR--D-----D

Llitoralis -------TLSARIPAGERAMTIPVDAISGVSGLLRPGDRVDLQNVTLLATLSLTPSEAELLVISQT----RGKLM-LLLRHR--D-----D

Pcaeni -------NLSSKIPPGERAMSIPVDNISGVSGLLRPGDRVDLQNVTLLATASVTIDEAELLTIAQT----RGELM-LLLRHN--E-----D

Bsediminis -------TLSSRIPPGERAMSLPVDTISGVSGLLRPGDRVDLQSVTLLATPSVTISEAELLTIAQT----RGELM-ILLRNS--E-----D

: . . .. :* : :: :
