# Supplementary material for: A Tad-like apparatus is required for contact-dependent prey killing in predatory social bacteria
Source: eLife. 2021 Sep 10;10:e72409. doi: 10.7554/eLife.72409 (PMC8460266; doi:10.7554/eLife.72409)
Supplement: Supplementary file 1. [file elife-72409-supp1.docx]

**Table 1: Characteristics of *Myxococcus xanthus* proteins**

| *M. xanthus* locus | Kill system | Function | Domain (InterPro) | Prediction method |
| --- | --- | --- | --- | --- |
| MXAN_RS15035  (MXAN_3102) |  | Unknown function | Cytochrome cd1 nitrite reductase | Bs : Proteome-BLAST |
| MXAN_RS15040 (MXAN_3103) |  | Unknown function | YWTD domain |  |
| MXAN_RS15045 (MXAN_3104) |  | Unknown function | FHA domain |  |
| MXAN_RS15050 (MXAN_3105) | KilA | Prepilin peptidase | Prepilin type IV endopeptidase, peptidase domain | Bb : domain comparison Cv : Proteome-BLAST  Bs : Proteome-BLAST |
| MXAN_RS15055 (MXAN_3106) | KilC | Secretin | BON domain ; Pilus formation protein, N-terminal ; secretin | Bb : Proteome-BLAST  Cv : Proteome-BLAST  Bs : Proteome-BLAST |
| MXAN_RS15060 (MXAN_3107) | KilF | ATPase | FHA domain ; P-loop containing nucleoside triphosphate hydrolase | Bb : Proteome-BLAST  Cv : Proteome-BLAST  Bs : Proteome-BLAST |
| MXAN_RS15065 (MXAN_3108) | KilD | Cytoplasmic multidomain protein | FHA domain ; Tetratricopeptide repeat | Bb : HHPRED |
| MXAN_RS22520 (MXAN_4639) |  | Unknown function | Tetratricopeptide repeat | Cv : Proteome-BLAST |
| MXAN_RS22565(MXAN_4648) |  | Protein kinase G 1.6E-18 | FHA domain ; Tetratricopeptide repeat | Bs : Proteome-BLAST |
| MXAN_RS22570 (MXAN_4649) |  | Type III secretion system protein PrgH-EprH (PrgH) 4.1E-10 | FHA domain | Bs : operon- BLAST |
| MXAN_RS22575  (MXAN_4650) | KilH | Inner-membrane platform protein | GspF domain | Bb : Proteome-BLAST  Cv : Proteome-BLAST Bs : operon- BLAST |
| MXAN_RS22580 (MXAN_4651) | KilG | Inner-membrane platform protein | GspF domain | Bb : Proteome-BLAST  Cv : Proteome-BLAST Bs : operon- BLAST |
| MXAN_RS22585 (MXAN_4652) | KilB | Outer-membrane protein, pilus assembly | SAF domain ; RcpC/CpaB domain | Bb : Proteome-BLAST  Cv : Proteome-BLAST  Bs : Proteome-BLAST |
| MXAN_RS22590(MXAN_4653) |  | Unknown function | X |  |
| MXAN_RS22595(MXAN_4654) |  | Unknown function | X |  |
| MXAN_RS22600 (MXAN_4655) | KilK | Pilin | X | Bb : operon- BLAST |
| MXAN_RS22605 (MXAN_4656) |  | Unknown function | X |  |
| MXAN_RS22610 (MXAN_4657) |  | Unknown function | X |  |
| MXAN_RS22615 (MXAN_4658) | KilL | Minor pilin | X | Bb : HHPRED  Cv : Proteome-BLAST |
| MXAN_RS22620 (MXAN_4659) |  | Unknown function | Putative Flp pilus-assembly TadG-like, N-terminal |  |
| MXAN_RS22625 (MXAN_4660) | KilM | Minor pilin | X | Bb : HHPRED Bs : Proteome-BLAST |
| MXAN_RS22630 (MXAN_4661) |  | Unknown function | Putative zinc-finger |  |

HHPRED
