# Supplementary material for: A Tad-like apparatus is required for contact-dependent prey killing in predatory social bacteria
Source: eLife. 2021 Sep 10;10:e72409. doi: 10.7554/eLife.72409 (PMC8460266; doi:10.7554/eLife.72409)
Supplement: Supplementary file 2. [file elife-72409-supp2.docx]

**Table 2: *Myxococcus xanthus* proteins with homologs identified in *Bdellovibrio bacteriovorus* HD100, *Caulobacter crescentus* CB15 and *Bradymonas sediminis***

| *M. xanthus* locus | Kill system | *Bdellovibrio* locus | Search method | RBH, E-value | *Caulobacter* locus | Search method | RBH, E-value | *Bradymonas* locus | Search method | RBH, E-value |
| --- | --- | --- | --- | --- | --- | --- | --- | --- | --- | --- |
| MXAN_RS15050 (MXAN_3105) | KilA | BD_RS00540 (Bd0114, hypothetical protein) | operon-BLAST, domain comparison | conserved domain architecture | CC_2947 (pilus assembly protein CpaA) | Proteome-BLAST | remote homology, paralog, 9.8 / 9.4 | DN745_18205 (hypothetical protein) ⇔ prepilin peptidase | Proteome-BLAST | ortholog, 4e-8/1e-7 |
| MXAN_RS15055 (MXAN_3106) | KilC | BD_RS00530 (Bd0112, BON domain-containing protein (Flp pilus assembly protein secretin CpaC)) | Proteome-BLAST | paralog, 1e-18 / 4e-19 | CC_2945 (pilus assembly protein CpaC) | Proteome-BLAST | ortholog, 1e-27 / 1e-28 | DN745_00685  (Flp pilus assembly secretin CpaC) | Proteome-BLAST | ortholog,8e-19/7e-31 |
| MXAN_RS15060 (MXAN_3107) | KilF | BD_RS00525 (Bd0111, P-loop NTPase (Flp pilus assembly protein ATPase CpaF)) | Proteome-BLAST | ortholog, 1e-152 / 2e-160 | CC_2942 (pilus assembly protein CpaF) | Proteome- BLAST | ortholog, 7e-130 / 9e-140 | DN745_00690  ⇔ Flp pilus assembly complex ATPase component TadA | Proteome- BLAST | ortholog, 5e-153/3e-143 |
| MXAN_RS15065 (MXAN_3108) | KilD |  |  |  |  |  |  |  |  |  |
| MXAN_RS22575 (MXAN_4650) | KilH | BD_RS02160 (Bd0470, type II secretion system F family protein) | Proteome-BLAST | ortholog, 3e-45 / 8e-45 | CC_2940 (hypothetical protein) | Proteome-BLAST | ortholog, 1e-23 / 4e-24 | DN745_00700 (hypothetical | operon-BLAST | 1e-31 |
| MXAN_RS22580 (MXAN_4651) | KilG | BD_RS00520 (Bd0110, Flp pilus assembly protein TadB) | Proteome-BLAST | ortholog, 1e-40 / 4e-41 | CC_2941 (hypothetical protein) | Proteome-BLAST | ortholog, 2e-23 / 8e-23 | DN745_00695 ( hypothetical | operon-BLAST | 1e-42 |
| MXAN_RS22585 (MXAN_4652) | KilB | BD_RS00535 (Bd0113, Flp pilus assembly protein CpaB) | Proteome-BLAST | ortholog, 2e-10 / 1e-11 | CC_2946 (pilus assembly protein CpaB) | Proteome-BLAST | ortholog, 9e-08 / 2e-07 | DN745_00680 (Flp pilus assembly protein CpaB) | Proteome-BLAST | ortholog, 3e-22/1e-20 |
| MXAN_RS22600 (MXAN_4655) | KilK | BD_RS00550 (Bd0117); BD_RS00560 (Bd0119); | operon- BLAST | remote homology  potential flp-protein |  |  |  |  |  |  |
| MXAN_RS22615 (MXAN_4658) | KilL | BD_RS00545 (Bd0115, hypothetical protein) | operon-BLAST; HHPRED | remote homology, 3.6e-6 | CC_2951 (hypothetical protein) | Proteome-BLAST | remote homology, uni-directional,  10.0 |  |  |  |
| MXAN_RS22625 (MXAN_4660) | KilM | BD_RS00545 (Bd0115, hypothetical protein) | HHPRED | remote homology  1.9e-12 |  |  |  | DN745_18665  (TadE-like protein) | Proteome- BLAST | ortholog, 1e-3/3e-3 |
