# Supplementary material for: A Tad-like apparatus is required for contact-dependent prey killing in predatory social bacteria
Source: eLife. 2021 Sep 10;10:e72409. doi: 10.7554/eLife.72409 (PMC8460266; doi:10.7554/eLife.72409)
Supplement: Supplementary file 3. [file elife-72409-supp3.docx]

| **Code** | **Strain** | **Genetic background** | **Source** |
| --- | --- | --- | --- |
| **TM108** | DZ2 | WT | Laboratory collection |
| **TM146** | DZ2 Δ*aglQ* | Chromosomal deletion of *aglQ* | Laboratory collection |
| **TM294** | DZ2 Ω*pilA* | DZ2 *pilA*::tet | Laboratory collection |
| **TM389** | DZ2 ∆*pilA* | Chromosomal deletion of *pilA* | Laboratory collection |
| **TM357** | DZ2 ∆*pilA* Δ*aglQ* | Chromosomal deletion of *pilA* and *aglQ* | Laboratory collection |
| **TM615** | DZ2 ∆*pilA* IMss mCherry | DZ2 ∆*pilA* transformed with pSWU19 *PpilA*-IMss-mCherry | Laboratory collection |
| **TM617** | DZ2 IMss mCherry | DZ2 WT transformed with pSWU19 *PpilA*-IMss-mCherry | Laboratory collection |
| **TM685** | DZ2 OMss sfGFP | DZ2 transformed with pSWU19 *PpilA*-OMss-sfGFP | Laboratory collection |
| **TM1123** | DZ2 ΔT6SS | Chromosomal deletion of T6SS | Laboratory collection |
| **TM1180** | DZ2 VipA-GFP | Allelic replacement of *vipA* by *vipA-GFP* | This work |
| **TM1194** | DZ2 Δ*aglQ* IMss mCherry | DZ2 Δ*aglQ* transformed with pSWU19 *PpilA*-IMss-mCherry, Kan^R^ | This work |
| **TM1297** | DZ2 AglZ Neon green | Allelic replacement of *aglZ* by *aglZ-NG* | This work |
| **TM1343** | DZ2 Δ3105-3107 (∆*kilACF*) OMss sfGFP | DZ2 Δ3105-3107 (∆*kilACF*) transformed with pSWU19 *PpilA*-OMss-sfGFP, Kan^R^ | This work |
| **TM1355** | DZ2 Δ3105-3107 (∆*kilACF*) | Chromosomal deletion of Mxan_3105-Mxan_3107 (∆*kilACF*) | This work |
| **TM1376** | DZ2 Δ3105 (∆*kilA*) | Chromosomal deletion of Mxan_3105 (∆*kilA*) | This work |
| **TM1377** | DZ2 Δ3107 (∆*kilF*) | Chromosomal deletion of Mxan_3107 (∆*kilF*) | This work |
| **TM1378** | DZ2 Δ3108 (∆*kilD*) | Chromosomal deletion of Mxan_3108 (∆*kilD*) | This work |
| **TM1379** | DZ2 Δ4650 (∆*kilH*) | Chromosomal deletion of Mxan_4650 (∆*kilH*) | This work |
| **TM1385** | DZ2 Δ4650 (∆*kilH*) OMss sfGFP | DZ2 Δ4650 (*kilH*) transformed with pSWU19 *PpilA*-OMss-sfGFP, Kan^R^ | This work |
| **TM1386** | DZ2 Δ4655 (∆*kilK*) | Chromosomal deletion of Mxan_4655 (∆*kilK*) | This work |
| **TM1388** | DZ2 Δ3106 (∆*kilC*) | Chromosomal deletion of Mxan_3106 (∆*kilC*) | This work |
| **TM1389** | DZ2 Neon green 3108 (∆*kilD*) | Allelic replacement of *kilD* by *NG*-*kilD* (Mxan_3108) | This work |
| **TM1390** | DZ2 Δ4658 (∆*kilL*) | Chromosomal deletion of Mxan_4658 (∆*kilL*) | This work |
| **TM1391** | DZ2 Δ4660 (∆*kilM*) | Chromosomal deletion of Mxan_4660 (∆*kilM*) | This work |
| **TM1398** | DZ2 Δ3106 (∆*kilC*) Neon green 3108 (NG-*kilD*) | Allelic replacement of *kilD* by *NG*-*kilD* (Mxan_3108) in DZ2 ΔMxan_3106 (∆*kilC*) | This work |
| **TM1399** | DZ2 Δ4650 (∆*kilH)* Neon green 3108 (NG-*kilD*) | Allelic replacement of *kilD* by *NG*-*kilD* (Mxan_3108) in DZ2 ΔMxan_4650 (∆*kilH*) | This work |
| **TM1402** | DZ2 Δ4655 (∆*kilB*) Neon green 3108 (NG-*kilD*) | Allelic replacement of *kilD* by *NG*-*kilD* (Mxan_3108) in DZ2 ΔMxan_4655 (∆*kilB*) | This work |
| **TM1404** | DZ2 Δ4655 Δ4658 (∆*kilKL*) | Chromosomal deletion of Mxan_4658 (∆*kilL*) in DZ2 ΔMxan_4655 (∆*kilK*) | This work |
| **TM1406** | DZ2 Δ4655 Δ4658 Δ4660 (∆*kilKLM*) | Chromosomal deletion of Mxan_4660 (∆*kilM*) in DZ2 ΔMxan_4655 ΔMxan_4658 (∆*kilKL*) | This work |
| **TM1415** | DZ2 Δ3106 (∆*kilC*) OMss sfGFP | DZ2 Δ3106 (∆*kilC*) transformed with pSWU19 *PpilA*-OMss-sfGFP, Kan^R^ | This work |
| **TM1416** | DZ2 Δ3107 (∆*kilF*) OMss sfGFP | DZ2 Δ3107 (∆*kilF*) transformed with pSWU19 *PpilA*-OMss-sfGFP, Kan^R^ | This work |
| **TM1418** | DZ2 Δ4655 (∆*kilK*) OMss sfGFP | DZ2 Δ4655 (∆*kilK*) transformed with pSWU19 *PpilA*-OMss-sfGFP, Kan^R^ | This work |
| **TM1422** | DZ2 Δ4655 Δ4658 Δ4660 (∆*kilKLM*) OMss sfGFP | DZ2 Δ4655 Δ4658 Δ4660 (∆*kilKLM*) transformed with pSWU19 *PpilA*-OMss-sfGFP, Kan^R^ | This work |
| **TM1433** | DZ2 Δ4651 (∆*kilG*) | Chromosomal deletion of Mxan_4651 (∆*kilG*) | This work |
| **TM1435** | DZ2 Δ4652 (∆*kilB*) | Chromosomal deletion of Mxan_4652 (∆*kilB*) | This work |
| **TM1437** | DZ2 Δ4651 (∆*kilG*) Neon green 3108 (NG-*kilD*) | Allelic replacement of *kilD* by *NG*-*kilD* (Mxan_3108) in DZ2 ΔMxan_4651 (∆*kilG*) | This work |
| **TM1439** | DZ2 Δ4652 (∆*kilB*) Neon green 3108 (NG-*kilD*) | Allelic replacement of *kilD* by *NG*-*kilD* (Mxan_3108) in DZ2 ΔMxan_4652 (∆*kilB*) | This work |
| **TM1440** | DZ2 Δ4655 Δ4658 Δ4660 (*kilKLM*) Neon green 3108 (NG *kilD*) | Allelic replacement of *kilD* by *NG*-*kilD* (Mxan_3108) in DZ2 ΔMxan_4655 ΔMxan_4658 ΔMxan_4660 (∆*kilKLM*) | This work |
| **TM1481** | DZ2 Neon Green 3107 (NG-*kilF*) | Allelic replacement of *kilF* by *NG*-*kilF* (Mxan_3107) | This work |
| **TM1480** | DZ2 WT pSWU19 *PpilA* empty vector | DZ2 WT transformed with pSWU19 *PpilA* empty vector, Kan^R^ | This work |
| **TM1482** | DZ2 ∆3106 (∆*kilC*) pSWU19 *PpilA*-*kilC* | DZ2 Δ3106 (∆*kilC*) transformed with pSWU19 *PpilA*-*kilC* (Mxan_3106) for complementation | This work |
| **TM1483** | DZ2 ∆3106 (∆*kilC*) pSWU19 *PpilA* empty vector | DZ2 Δ3106 (∆*kilC*) transformed with pSWU19 *PpilA*-EV (empty vector) | This work |
| **TM1484** | DZ2 ∆3107 (∆*kilF*) pSWU19 *PpilA*-*kilF* | DZ2 Δ3107 (∆*kilF*) transformed with pSWU19 *PpilA*-*kilF* (Mxan_3107) for complementation | This work |
| **TM1485** | DZ2 ∆3107 (∆*kilF*) pSWU19 *PpilA* empty vector | DZ2 Δ3107 (∆*kilC*) transformed with pSWU19 *PpilA*-EV (empty vector) | This work |
| **TM1486** | DZ2 ∆4650 (∆*kilH*) pSWU19 *PpilA*-*kilH* | DZ2 Δ4650 (∆*kilH*) transformed with pSWU19 *PpilA*-*kilH* (Mxan_4650) for complementation | This work |
| **TM1487** | DZ2 ∆4650 (∆*kilH*) pSWU19 *PpilA* empty vector | DZ2 Δ4650 (∆*kilH*) transformed with pSWU19 *PpilA*-EV (empty vector) | This work |
| **TM1488** | DZ2 ∆4651 (∆*kilG*) pSWU19 *PpilA*-*kilG* | DZ2 Δ4651 (∆*kilG*) transformed with pSWU19 *PpilA*-*kilG* (Mxan_4651) for complementation | This work |
| **TM1489** | DZ2 ∆4651(∆*kilG*) pSWU19 *PpilA* empty vector | DZ2 Δ4651 (∆*kilG*) transformed with pSWU19 *PpilA*-EV (empty vector) | This work |
| **TM1490** | DZ2 ∆4651(∆*kilG*) pSWU19 *PpilA*-*kilG-NG* | DZ2 Δ4651 (∆*kilG*) transformed with pSWU19 *PpilA*-*kilG-NG* (Mxan_4651) | This work |
| **TM980** | DZ2 ∆*dacB* | Chromosomal deletion of *dacB* | Laboratory collection |

| **Code** | **Strain** | **Source** |
| --- | --- | --- |
| **EC393** | *Escherichia coli* MG1655 | Laboratory collection |
| **EC480** | *Bacillus subtilis* WT168 | Anne Galinier |
| **EC481** | *Pseudomonas aeruginosa* PAO1 | Sophie Bleves |
| **EC510** | *Escherichia coli* MG1655 GFP | This work |
| **EC511** | *Escherichia coli* MG1655 mCherry | This work |
| **EC525** | *Salmonella enterica* *Typhimurium* LT2 | Eric Cascales |
| **EC729** | *Caulobacter crescentus* NA1000 | Emmanuele Biondi |
| **EC397** | *Escherichia coli* MG1655 kanamycin resistant | Laboratory collection |
| **EC799** | *Salmonella enterica* *Typhimurium* LT2 kanamycin resistant | Eric Cascales |
| **EC800** | *Bacillus subtilis* WT168 kanamycin resistant | Anne Galinier |
| **EC801** | *Caulobacter crescentus* NA1000 kanamycin resistant | Emmanuele Biondi |
