# Supplementary material for: A Tad-like apparatus is required for contact-dependent prey killing in predatory social bacteria
Source: eLife. 2021 Sep 10;10:e72409. doi: 10.7554/eLife.72409 (PMC8460266; doi:10.7554/eLife.72409)
Supplement: Supplementary file 4. [file elife-72409-supp4.docx]

| **Plasmid name** | **Description** | **Source** |
| --- | --- | --- |
| pBJ114 | Used to create deletions, galK, Km^R^ | Laboratory collection |
| pSWU19 | Km^R^, used to integrate genes ectopically at Mx8 att | Laboratory collection |
| pBJ114 Δ3105 (*kilA*) | Suicide plasmid used for deletion of Mxan_3105 (*kilA*) by double recombination | This work |
| pBJ114 Δ3106 (*kilC*) | Suicide plasmid used for deletion of Mxan_3106 (*kilC*) by double recombination | This work |
| pBJ114 Δ3107 (*kilF*) | Suicide plasmid used for deletion of Mxan_3107 (*kilF*) by double recombination | This work |
| pBJ114 Δ3105-3107 (*kilACF*) | Suicide plasmid used for deletion of Mxan_3105-3107 (*kilACF*) by double recombination | This work |
| pBJ114 Δ3108 (*kilD*) | Suicide plasmid used for deletion of Mxan_3108 (*kilD*) by double recombination | This work |
| pBJ114 neonGreen-3108 (*kilD*) | Suicide plasmid used for replacing *kilD* with a neonGreen fusion by double recombination | This work |
| pBJ114 neonGreen-3107 (*kilF*) | Suicide plasmid used for replacing *kilF* with a neonGreen fusion by double recombination | This work |
| pBJ114 Δ4650 (*kilH*) | Suicide plasmid used for deletion of Mxan_4650 (*kilH*) by double recombination | This work |
| pBJ114 Δ4651 (*kilG*) | Suicide plasmid used for deletion of Mxan_4651 (*kilG*) by double recombination | This work |
| pBJ114 Δ4652 (*kilB*) | Suicide plasmid used for deletion of Mxan_4652 (*kilB*) by double recombination | This work |
| pBJ114 Δ4655 (*kilK*) | Suicide plasmid used for deletion of Mxan_4655 (*kilK*) by double recombination | This work |
| pBJ114 Δ4658 (*kilL*) | Suicide plasmid used for deletion of Mxan_4658 (*kilL*) by double recombination | This work |
| pBJ114 Δ4660 (*kilM*) | Suicide plasmid used for deletion of Mxan_4660 (*kilM*) by double recombination | This work |
| pBJ114 VipA-GFP | Suicide plasmid used for replacing VipA by VipA-GFP at the locus | Anke Treuner Lange |
| pSWU19 pPilA-IMss-mCherry | Used for ectopic expression of mCherry fused to an inner membrane signal sequence | Laboratory collection |
| pSWU19 pPilA-OMss-sfGFP | Used for ectopic expression of sfGFP fused to an outer membrane signal sequence | Laboratory collection |
| pCBP-GFP::km | Used for expression of GFP in the cytoplasm of *E. coli* | Laurent Aussel |
| pGG2-rpsm-mcherry | Used for expression of mCherry in the cytoplasm of *E. coli* | Laurent Aussel |
| pSWU19 *PpilA* empty vector | Used for ectopic expression (at Mx8 att) of the *kil* genes under the control of the *pilA* promoter | This work |
| pSWU19 *PpilA*-3106 (*kilC*) | Used for ectopic expression of *kilC* (Mxan_3106) under the control of the *pilA* promoter | This work |
| pSWU19 *PpilA*-3107 (*kilF*) | Used for ectopic expression of *kilF* (Mxan_3107) under the control of the *pilA* promoter | This work |
| pSWU19 *PpilA*-4650 (*kilH*) | Used for ectopic expression of *kilH* (Mxan_4650) under the control of the *pilA* promoter | This work |
| pSWU19 *PpilA*-4651 (*kilG*) | Used for ectopic expression of *kilG* (Mxan_4651) under the control of the *pilA* promoter | This work |
| pSWU19 *PpilA*-4651-neonGreen (*kilG*) | Used for ectopic expression of *kilG-NG* (Mxan_4651 fused to Neon Green) under the control of the *pilA* promoter | This work |

Table 4 : plasmids
