# Supplementary material for: A Tad-like apparatus is required for contact-dependent prey killing in predatory social bacteria
Source: eLife. 2021 Sep 10;10:e72409. doi: 10.7554/eLife.72409 (PMC8460266; doi:10.7554/eLife.72409)
Supplement: Supplementary file 5. [file elife-72409-supp5.docx]

| **Name** | **Primer sequence (5'-3')** | **Construction** |
| --- | --- | --- |
| oss104 (Δ3105 fw1) | ACAGCTATGACATGATTACAAGCTTGCCGCCGTGGCACCCGTGGC | Δ3105 (*kilA*) in pBJ114 |
| oss105 (Δ3105 rv 1) | CCCTCTTCTCTTCCAGAAGTCCGAGCGCCTGTCCTCTTCGCGGCA | Δ3105 (*kilA*) in pBJ114 |
| oss106 (Δ3105 fw 2) | ACGCCTGCCGCGAAGAGGACAGGCGCTCGGACTTCTGGAAGAGAA | Δ3105 (*kilA*) in pBJ114 |
| oss107 (Δ3105 rv 2) | TAAAACGACGGCCAGTGCCGAATTCCCCCGTGATGTCCGTCGGGT | Δ3105 (*kilA*) in pBJ114 |
| oss111 (Δ3106 fw 1) | ACAGCTATGACATGATTACAAGCTTAGACCTGGGCTCGTCCAACG | Δ3106 (*kilC*) in pBJ114 |
| oss112 (Δ3106 rv 1) | AGGCGGCGCCCCAGGGGCGCCCACCCGTCCGTGTCCCCTCTTCTC | Δ3106 (*kilC*) in pBJ114 |
| oss113 (Δ3106 fw 2) | TGGAAGAGAAGAGGGGACACGGACGGGTGGGCGCCCCTGGGGCGC | Δ3106 (*kilC*) in pBJ114 |
| oss114 (Δ3106 rv 2) | TAAAACGACGGCCAGTGCCGAATTCATGGCCTTCTCGGTGCGGCG | Δ3106 (*kilC*) in pBJ114 |
| oss118 (Δ3107 fw 1) | ACAGCTATGACATGATTACAAGCTTTCGGCTCCGGCGTTTCCACG | Δ3107 (*kilF*) in pBJ114 |
| oss119 (Δ3107 rv 1) | GACGACCAGGGTGGCCATGGGCGCGGGGGACGGATGCTAGCAAGC | Δ3107 (*kilF*) in pBJ114 |
| oss120 (Δ3107 fw 2) | GGGGGGCTTGCTAGCATCCGTCCCCCGCGCCCATGGCCACCCTGG | Δ3107 (*kilF*) in pBJ114 |
| oss121 (Δ3107 rv 2) | TAAAACGACGGCCAGTGCCGAATTCAGGTCATCTCCACCACGCCG | Δ3107 (*kilF*) in pBJ114 |
| oss70 (Δ3108 fw 1) | ACAGCTATGACATGATTACAAGCTTTCCCTGCATCACCATCCGCA | Δ3108 (*kilD*) in pBJ114 |
| oss71 (Δ3108 rv 1) | CGCCCGGCCCTGTCCACGACGCGTGGGGCGCGTCAGTCCTCGCGG | Δ3108 (*kilD*) in pBJ114 |
| oss72 (Δ3108 fw 2) | AGGACTGACGCGCCCCACGCGTCGTGGACAGGGCCGGGCGGGCGC | Δ3108 (*kilD*) in pBJ114 |
| oss73 (Δ3108 rv 2) | TAAAACGACGGCCAGTGCCGAATTCGTCTTGAAGGTGACGAGCAC | Δ3108 (*kilD*) in pBJ114 |
| oss27 (Δ3105-07 fw 1) | ACAGCTATGACATGATTACAAGCTTGAGGACGAGGAGCCGCTGCC | Δ3105-07 (*kilACF*) in pBJ114 |
| oss28 (Δ3105-07 rv 1) | GCTCGGTACCCGGGGATCCTCTAGACGCCACCCGGGGCGGGACGT | Δ3105-07 (*kilACF*) in pBJ114 |
| oss29 (Δ3105-07 fw 2) | TGCCGCGAAGAGGACAGGCGCGCGCCCATGGCCACCCTGGTCGTC | Δ3105-07 (*kilACF*) in pBJ114 |
| oss30 (Δ3105-07 rv 2) | GACGACCAGGGTGGCCATGGGCGCGCGCCTGTCCTCTTCGCGGCA | Δ3105-07 (*kilACF*) in pBJ114 |
| oss175 (Δ4650 fw 1) | ACAGCTATGACATGATTACAAGCTTTCGGCTTCTTCCTGCCGATG | Δ4650 (*kilH*) in pBJ114 |
| oss176 (Δ4650 rv 1) | TGGTCAGGAGGATGGGCACGGGGCGGGTGCACCTCTCAGATATCG | Δ4650 (*kilH*) in pBJ114 |
| oss177 (Δ4650 fw 2) | AACATCGATATCTGAGAGGTGCACCCGCCCCGTGCCCATCCTCCT | Δ4650 (*kilH*) in pBJ114 |
| oss178 (Δ4650 rv 2) | TAAAACGACGGCCAGTGCCGAATTCTAGCTGCCCCCACCACAGGA | Δ4650 (*kilH*) in pBJ114 |
| JH Δ4651 fw 1 | TATGACATGATTACAAGCTTGTCATCGCGTATTCCGCCAT | Δ4651 (*kilG*) in pBJ114 |
| JH Δ4651 rv 1 | GCGCCGGATGATGAGGATGCCTGGATCGATGAAGAGGAACAT | Δ4651 (*kilG*) in pBJ114 |
| JH Δ4651 fw 2 | ATGTTCCTCTTCATCGATCCAGGCATCCTCATCATCCGGCGC | Δ4651 (*kilG*) in pBJ114 |
| JH Δ4651 rv 2 | CGACGGCCAGTGCCGAATTCTCACCGGCGCCTCGCCGGCC | Δ4651 (*kilG*) in pBJ114 |
| JH Δ4652 fw 1 | TATGACATGATTACAAGCTTGGCCTGGGCGAGGACGACGG | Δ4652 (*kilB*) in pBJ114 |
| JH Δ4652 rv 1 | TCACGGCGCGCCAGCGCCCGCCGGAGTCTTGCCCTTCAGCAT | Δ4652 (*kilB*) in pBJ114 |
| JH Δ4652 fw 2 | ATGCTGAAGGGCAAGACTCCGGCGGGCGCTGGCGCGCCGTGA | Δ4652 (*kilB*) in pBJ114 |
| JH Δ4652 rv 2 | CGACGGCCAGTGCCGAATTCGATGTGGTCCATCATCGGCTC | Δ4652 (*kilB*) in pBJ114 |
| oss153 (Δ4655 fw 1) | ACAGCTATGACATGATTACAAGCTTGGGCCGGGACGCGGTGTGGG | Δ4655 (*kilK*) in pBJ114 |
| oss154 (Δ4655 rv 1) | GGCTGCCCCACCTCACTGCTTGCCGGGCGTGTCCTCAGAGGTGTC | Δ4655 (*kilK*) in pBJ114 |
| oss155 (Δ4655 fw 2) | GACACGACACCTCTGAGGACACGCCCGGCAAGCAGTGAGGTGGGG | Δ4655 (*kilK*) in pBJ114 |
| oss156 (Δ4655 rv 2) | TAAAACGACGGCCAGTGCCGAATTCCCACGCCATCCGGCGTCACG | Δ4655 (*kilK*) in pBJ114 |
| oss201 (Δ4658 fw 1) | ACAGCTATGACATGATTACAAGCTTCGGCCGGGTGGCCCCGAGCG | Δ4658 (*kilL*) in pBJ114 |
| oss202 (Δ4658 rv 1) | AAGTGCCGTCCGGGCGGCCACCACCGGCAGTCCTCAGTGCGTCAT | Δ4658 (*kilL*) in pBJ114 |
| oss203 (Δ4658 fw 2) | AGATCATGACGCACTGAGGACTGCCGGTGGTGGCCGCCCGGACGG | Δ4658 (*kilL*) in pBJ114 |
| oss204 (Δ4658 rv 2) | TAAAACGACGGCCAGTGCCGAATTCTGACCCCCTCATCCGTGGGC | Δ4658 (*kilL*) in pBJ114 |
| oss194 (Δ4660 fw 1) | ACAGCTATGACATGATTACAAGCTTGTCGGAGCGGCGCGTCGCGG | Δ4660 (*kilM*) in pBJ114 |
| oss195 (Δ4660 rv 1) | ACATGGGTCGGGAGTCCTCTTCCTCGAAACCATTATCCGGGGACT | Δ4660 (*kilM*) in pBJ114 |
| oss196 (Δ4660 fw 2) | ACACCAGTCCCCGGATAATGGTTTCGAGGAAGAGGACTCCCGACC | Δ4660 (*kilM*) in pBJ114 |
| oss197 (Δ4660 rv 2) | TAAAACGACGGCCAGTGCCGAATTCATGACCGTCTGCGTGGTCTG | Δ4660 (*kilM*) in pBJ114 |
| oss188 (Neon green 3108 fw 1) | ACAGCTATGACATGATTACAAGCTTCGGGAAGACGACGACGCTGA | Neon green 3108 (*kilD*) in pBJ114 |
| oss189 (Neon green 3108 rv 1) | TATCCTCCTCTCCTTTCGAAACCATGGGCGCGTCAGTCCTCGCGG | Neon green 3108 (*kilD*) in pBJ114 |
| oss190 (Neon green 3108 fw 2) | ATCTTCCGCGAGGACTGACGCGCCCATGGTTTCGAAAGGAGAGGA | Neon green 3108 (*kilD*) in pBJ114 |
| oss191 (Neon green 3108 rv 2) | GGTGACGGACGACCAGGGTGGCCATACCTGAGCCGCTTCCTGAAC | Neon green 3108 (*kilD*) in pBJ114 |
| oss192 (Neon green 3108 fw 3) | CTCAGGTTCAGGAAGCGGCTCAGGTATGGCCACCCTGGTCGTCCG | Neon green 3108 (*kilD*) in pBJ114 |
| oss193 (Neon green 3108 rv 3) | TAAAACGACGGCCAGTGCCGAATTCTCCGCGTGGCGGCGGCTCAC | Neon green 3108 (*kilD*) in pBJ114 |
| JH PpilA 1000 bp region fw | ACGACGGCCAGTGCCAAGCTTATCCATATGCGCGGCACTCGAGCC | Cloning of *PpilA* in pSWU19 |
| JH PpilA 1000 bp region rv | GGGGGTCCTCAGAGAAGGTTGCAACG | Cloning of *PpilA* in pSWU19 |
| JH PpilA EV rv | ATGACCATGATTACGAATTCTCAGATGCGTGTGAACATGGGGGTCCTCAGAGAAGGTTGCAACG | Cloning of *PpilA* in pSWU19 (Empty vector control) |
| JH 3106_complementation fw | CGTTGCAACCTTCTCTGAGGACCCCCATGTTCACACGCATCACGCATGCCG | Cloning of *kilC* in pSWU19-*pPilA* |
| JH 3106_ complementation rv | ATGACCATGATTACGAATTCTCAGTCGAAGATGTTGAAGTTCACCTC | Cloning of *kilC* in pSWU19-*pPilA* |
| JH 3107_ complementation fw | CGTTGCAACCTTCTCTGAGGACCCCCATGTTTCTCATCACCCTCGCTGAAAAG | Cloning of *kilF* in pSWU19-*pPilA* |
| JH 3107_ complementation rv | ATGACCATGATTACGAATTCTCAGTCCTCGCGGAAGATGCTCATG | Cloning of *kilF* in pSWU19-*pPilA* |
| JH 4650_ complementation fw | CGTTGCAACCTTCTCTGAGGACCCCCATGGACGATGTCCTGACGCCAATCTTG | Cloning of *kilH* in pSWU19-*pPilA* |
| JH 4650_ complementation rv | ATGACCATGATTACGAATTCCTACCCCGCGACGTTTCCGAACATG | Cloning of *kilH* in pSWU19-*pPilA* |
| JH 4651_ complementation fw | CGTTGCAACCTTCTCTGAGGACCCCCATGCTTGCTGGAATCGTCCTCCTC | Cloning of *kilG* in pSWU19-*pPilA* |
| JH 4651_ complementation rv | ATGACCATGATTACGAATTCTCAGATATCGATGTTGACGATGCGC | Cloning of *kilG* in pSWU19-*pPilA* |
| JH 4651-linker-NG rev | TCCAGACCCAGAACGCACCTGAGCCCTTCCTGAGATATCGATGTTGACGATGCGCCG | Cloning of *kilG+linker-NG* in pSWU19-*pPilA* |
| JH linker-NG fw | TCAGGAAGGGCTCAGGTGCGTTCTGGGTCTGGAATGGTTTCGAAAGGAGAGGAGGATA | Cloning of *kilG+linker-NG* in pSWU19-*pPilA* |
| JH pSWU19-NG rev | ATGACCATGATTACGAATTCTCACTTATAGAGTTCATCCATACCCATC | Cloning of *kilG+linker-NG* in pSWU19-*pPilA* |
| JH NG-3107 fw 1 | ACAGCTATGACATGATTACAAGCTTTCGGCTCCGGCGTTTCCACG | Cloning of NeonGreen-linker-*kilF* in pBJ114 |
| JH NG-3107 rv 1 | TCCTCTCCTTTCGAAACCATGGGGACGGATGCTAGCAAGC | Cloning of NeonGreen-linker-*kilF* in pBJ114 |
| JH NG-3107 fw 2 | GCTTGCTAGCATCCGTCCCCATGGTTTCGAAAGGAGAGGA | Cloning of NeonGreen-linker-*kilF* in pBJ114 |
| JH NG-3107 rv 2 | TCAGCGAGGGTGATGAGAAACATACCTGAGCCGCTTCCTGAACCTGAG | Cloning of NeonGreen-linker-*kilF* in pBJ114 |
| JH NG-3107 fw 3 | CTCAGGTTCAGGAAGCGGCTCAGGTATGTTTCTCATCACCCTCGCTGA | Cloning of NeonGreen-linker-*kilF* in pBJ114 |
| JH NG-3107 rv 3 | TAAAACGACGGCCAGTGCCGAATTCTGGTTGGCGTGGTTCACCAT | Cloning of NeonGreen-linker-*kilF* in pBJ114 |
